# Fail-safe genetic codes designed to intrinsically contain engineered organisms

**DOI:** 10.1093/nar/gkz745

**Published:** 2019-09-12

**Authors:** Jonathan Calles, Isaac Justice, Detravious Brinkley, Alexa Garcia, Drew Endy

**Affiliations:** 1 Bioengineering Department, Stanford University, Stanford, CA 94305, USA; 2 Department of Mathematics and Computer Science, Claflin University, Orangeburg, SC 29115, USA

## Abstract

One challenge in engineering organisms is taking responsibility for their behavior over many generations. Spontaneous mutations arising before or during use can impact heterologous genetic functions, disrupt system integration, or change organism phenotype. Here, we propose restructuring the genetic code itself such that point mutations in protein-coding sequences are selected against. Synthetic genetic systems so-encoded should fail more safely in response to most spontaneous mutations. We designed fail-safe codes and simulated their expected effects on the evolution of so-encoded proteins. We predict fail-safe codes supporting expression of 20 or 15 amino acids could slow protein evolution to ∼30% or 0% the rate of standard-encoded proteins, respectively. We also designed quadruplet-codon codes that should ensure all single point mutations in protein-coding sequences are selected against while maintaining expression of 20 or more amino acids. We demonstrate experimentally that a reduced set of 21 tRNAs is capable of expressing a protein encoded by only 20 sense codons, whereas a standard 64-codon encoding is not expressed. Our work suggests that biological systems using rationally depleted but otherwise natural translation systems should evolve more slowly and that such hypoevolvable organisms may be less likely to invade new niches or outcompete native populations.

## INTRODUCTION

The ability to engineer organisms is increasingly important for academic, industrial and public uses (1–9). Traditional engineering disciplines have established methods for controlling systems on the timescales of immediate input and response (e.g. autonomous control) (10,11), and intermediate learning and memory (e.g. algorithms that learn) (12,13). However, living systems also perform on evolutionary timescales, realizing complicated behaviors across generations (1,14). To reliably operate engineered organisms capable of reproduction, we must learn to how to engineer lineages on evolutionary timescales (15).

Evolution within a population relies on the diversity of genetic makeups (i.e. genotypes) from which emerges a corresponding diversity of physiological and behavioral traits (i.e. phenotypes). Genetic diversity is most-often generated by error during DNA replication (i.e. mutation) and propagated across generations (16,17). Individuals with phenotypes better suited to a given environment tend to reproduce more successfully, enriching the population with their offspring (17–19). Thus, to engineer the evolutionary trajectories of individual organisms competing within populations, we must either control the processes that generate mutations or the selective pressures acting within and among populations (20).

One direct approach to controlling the behavior of engineered organisms over multiple generations is to reduce organism fitness outside of a prescribed niche. Scientists have long sought and realized such control of engineered organisms for the safe advancement of fundamental research (21,22). For example, biocontainment methods such as engineered auxotrophy (23–25) or exogenously expressed ‘kill signals’ (26–32) have been widely used. However, such methods can be detrimental to their host organisms and may result in selective pressures that inactivate the underlying mechanism (33).

More general approaches for controlling behavior over multiple generations consider altering the type and effect of mutations that arise. Such approaches generally take advantage of degeneracy in the mapping of DNA to proteins (i.e. the ‘genetic code’) to synonymously recode genes of interest (34). Recoding approaches work by altering the distribution of phenotypes available to an individual without changing the proteins expressed by that individual. For example, an organism can be recoded such that its initial fitness is high but nearby regions of its mutational space are less fit or even fatal. Such approaches have been tested by synonymously recoding Coxsackie B3 and influenza A viruses so that their genotypes were immediately adjacent to deleterious genotypes, resulting in attenuated virulence via decreased evolutionary rates (35). Another approach is to recode an organism so that its initial fitness is lowered and no single mutation results in a significant restoration of fitness; so-encoded organisms might be safely deployed for a limited number of generations. Such a strategy was tested by introducing infrequently used codon pairs into the poliovirus genome via synonymous recoding, resulting in both attenuated virulence and reduced likelihood of escape mutants arising during use (36). A third approach encodes an essential gene within the coding sequence of a gene of interest. Loss-of-function mutations in the essential gene are selected against, reducing the likelihood that the gene of interest is lost (Decrulle *et al.* 2019, preprint, 37). We note that while recoding- and overlap-based approaches are generalizable to most biological systems, only the local fitness landscape of an organism is affected; if an organism somehow escapes its local fitness trap then it can continue to evolve unimpeded.

**Figure 1.**
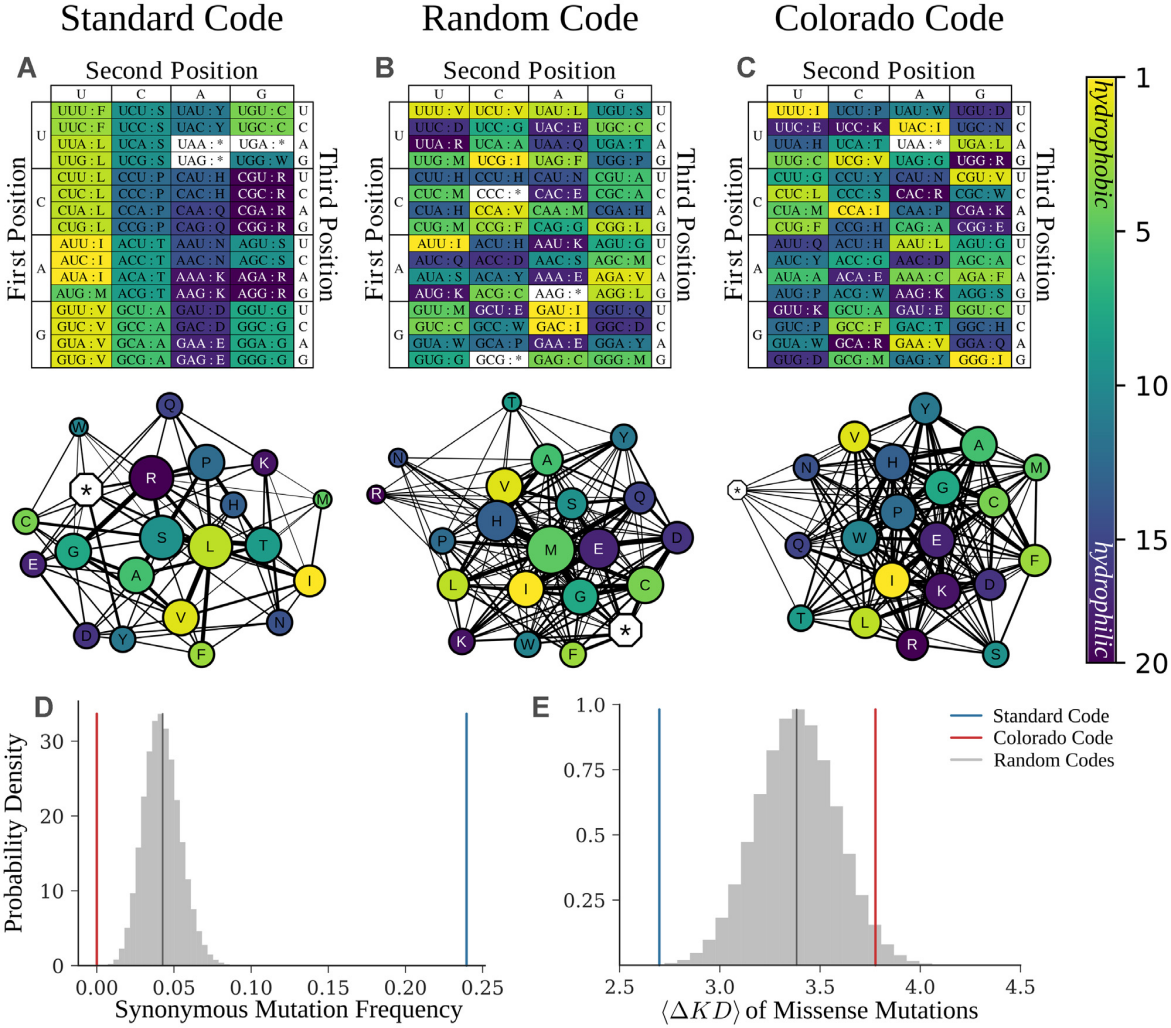
Genetic codes are expected to influence evolutionary dynamics. Table and mutation-distance network representations for the (**A**) Standard Code, (**B**) a genetic code with random structure, (**C**) and the Colorado Code. Color signifies the rank-ordered hydropathy of the amino acids—isoleucine (I) is most hydrophobic and arginine (R) is most hydrophilic. Mutation-distance networks represent amino acids as nodes. Node size represents the number of codons allocated to each amino acid or null. Edge weights between nodes (representing amino acids ${{\rm{a}}_1}$ and ${{\rm{a}}_2}$) represent the accessibility of ${{\rm{a}}_2}$ to ${{\rm{a}}_1}$ via point mutations. (**D**) Distribution of synonymous-mutation frequency (${{\rm{f}}_{\rm{s}}}$) for ${10^6}$ randomly generated codes (gray histogram) and mean of this distribution (black), as well as ${{\rm{f}}_{\rm{s}}}$ for the Standard Code (blue) and Colorado Code (red). (**E**) Distribution of mean mutation effects given a nonsynonymous mutation, ($\langle {\rm{\Delta KD}}\rangle$), for ${10^6}$ randomly generated codes (gray histogram) and mean of this distribution (black), as well as ${\rm{\Delta KD}}$ for the Standard Code (blue) and Colorado Code (red). We defined $\langle {\rm{\Delta KD}}\rangle$ of a genetic code as the average over all nonsynonymous mutations (from ${{\rm{a}}_1}$ to ${{\rm{a}}_2}$) of the change in Kyte–Doolittle hydropathy (${\rm{\Delta KD}}$) between the two residues (${\rm{\Delta KD\ }} = {\rm{\ }}| {{\rm{\ KD}}( {{{\rm{a}}_2}} ) - {\rm{KD}}( {{{\rm{a}}_1}} )} |$).

A more fundamental approach aims to control the entire fitness landscape of an organism by changing the underlying mapping of genotype to phenotype. Most known life uses the ‘Standard Code’ or a close variant thereof to assign 64 nucleotide triplets (i.e. ‘codons’) to 20 unique amino acids plus a termination signal (Figure 1A) (34,38). The Standard Code has a highly nonrandom structure that is optimized for translation fidelity across generations (Figure 1A and B) (34,39). For example, mutations in the Standard Code are significantly more likely than in a randomly generated code to conserve the encoded amino acid (24% vs. 4%), and to minimize the physicochemical change upon mutations that do not conserve the encoded amino acid (Figure 1D and E) (40). Redesigning the genetic code would alter the type and effect of spontaneous mutations across all genotypes, independent of the biological system using the code. For example, recent theoretical work by Pines and colleagues proposed a ‘hyperevolvable’ genetic code for use in directed evolution (hereafter ‘Colorado Code’) (41). More specifically, Pines *et al.* hypothesize that decreasing synonymous mutation likelihoods and increasing the physicochemical changes in amino acids resulting from missense mutations should result in greater phenotypic changes for each change in genotype (Figure 1C–E).

We propose the opposite—a set of ‘fail-safe’ genetic codes designed to map mutations to deleterious phenotypes independent of the biological system in which the fail-safe code is implemented. We designed a subset of such fail-safe codes so that they might be readily realized using natural translation machinery alone, avoiding the need for extensive molecular reengineering. We simulated the expected evolutionary dynamics of fail-safe encoded proteins in engineered organisms as well as the interaction between populations encoded using different genetic codes. We also implemented one such fail-safe code using a reduced set of 21 tRNA and found that the choosen code is capable of synthesizing proteins *in vitro*. Our results suggest that fail-safe codes might slow or altogether arrest the evolution of protein-coding sequences in fail-safe encoded organisms. Our results also suggest that fail-safe encoded organisms should be less able to compete with native species if introduced to new environmental contexts.

## MATERIALS AND METHODS

### Software

All code used herein is free online via https://github.com/EndyLab/codon-tables/tree/manuscript.

### Constructing mutation-distance networks

We made force-directed graphs to help understand the impact of point mutations in any given code. Nodes represent encoded amino acids and edges represent mutations between sense codons corresponding to those amino acids. Two nodes are connected by an edge if there exists at least one pair of codons, $c_1$ and $c_2$, encoding amino acids, $a_1$ and $a_2$, such that $c_1$ can be converted to $c_2$ by a single point mutation. The edge weight between any two amino acids ${{\rm{a}}_1}$ and ${{\rm{a}}_2}$ takes into account all possible acyclic paths between the set of codons encoding $a_1$ and $a_2$, respectively, including indirect paths that involve initial synonymous mutations. Individual paths from $c_1$ to ${{\rm{c}}_2}$ are weighted by an inverse power law representing the number of point mutations necessary to convert $c_1$ to $c_2$. Paths are then summed to give the total edge weight.

Formally, let $C = \{ \rm{UUU}, \ \ldots \ , \rm{GGG} \}$ be the set of all triplet codons, $A = \{ \rm{F}, \ \rm{L}, \ \ldots \ , \rm{G} \}$ be the set of all amino acids, $\mathbb{T}: c \to a \ | \ c \in C,\ a \in A$ be a genetic code, and $w( a_1, a_2)$ be the edge weight between amino acids $a_1$ and $a_2$:

$$\begin{equation*}w\ \left( {{a_1},\ {a_2}} \right) = \mathop \sum \limits_{{c_1}}^{{C_1}} \mathop \sum \limits_{{c_2}}^{{C_2}} {p^{l\left( {{c_1},\ {c_2}} \right)}}\delta \left( {{c_1},\ {c_2}} \right)\ \end{equation*}$$

Where ${C_i} = {\rm{\{ }}c \in C{\rm{\ |}}\ \ \mathbb{T}\ ( c ) = {a_i}{\rm{\ }}\} ,{\rm{\ }}l{\rm{\ }}( {{c_1},{\rm{\ }}{c_2}}) = {\rm{\ }}dist({{c_i},{\rm{\ }}{c_2}})$ (by how many nucleotides they differ), and

$$\begin{equation*}\delta \left( {{c_1},{\rm{\ }}{{\rm{c}}_2}} \right)\left\{ {\begin{array}{@{}*{1}{c}@{}} {1\ {\rm{if}}\ \exists \ \widetilde {{c_1}},\ \widetilde {{c_2}} \in C\ \left| {\begin{array}{@{}*{1}{c}@{}} {l\left( {\widetilde {{c_1}},\ \widetilde {{c_2}}} \right) \le 1,}\\ {\widetilde {{c_i}}\ {\rm{reachable\ from\ }}{c_i}{\rm{by\ synonymous\ mutation}}} \end{array}} \right.}\\ {0\ {\rm{otherwise}}}\end{array}}\right.,\end{equation*}$$

We used a value of 1/12 for the parameter $p \in$($0,\ 1)$ to scale edge weights by mutational distance.

### Modeling wobble decoding and tRNA promiscuity

When designing fail-safe codes we chose to decode sense codons using the tRNA species that would recognize the fewest additional codons. We also used the following heuristic rules: NNY codons (with U or C in the wobble position) can be decoded by tRNA species with anticodons GNN and QNN (where Q is queuosine); generally, tRNAs cannot discriminate NNU from NNC; similarly, NNR (with A or G in the wobble position) are decoded by tRNAs with modified uridine in the 34th position (e.g. cmnm5U, mcm5U, Um, and xm5s2U); while Ile-tRNA^CAU^ can distinguish AUA from AUG using k2C in the 34th position, this ability to decode NNA and not NNG does not generalize to all NNA decoding species; and NNG is fully distinguishable from all other codons with an unmodified C in the 34th position (42,43). A full description of our RNA base modification shorthand is provided (Supplementary Table S1).

### Simulating evolutionary dynamics

All simulations were carried out in Python 3.6.4 on Docker instances running Debian 8 hosted by Amazon Web Services (AWS). Parallelization was managed by AWS Batch. Each simulated strain was partitioned into one of two groups, based on population size, which were modeled independently over a small epoch ${\rm{dt}}$ (0.1 generations). We modeled small population-size groups using a stochastic birth-death model. The per-individual doubling probability in an epoch is given by ${{\rm{p}}_{\rm{b}}} = [ {1 + ( {{{\rm{f}}_{\rm{i}}} - \langle {\rm{f}}\rangle } )} ]{\rm{dt}}$ where ${{\rm{f}}_{\rm{i}}}$ is fitness of the *i*th strain and $\langle {\rm{f}}\rangle$ is the mean fitness of the population. The corresponding death probability is fixed at ${{\rm{p}}_{\rm{d}}} = ( 1 ){\rm{\ dt}}$. We modeled the large population-size group analytically with strain size ${{\rm{N}}_{\rm{i}}}$ given by ${{\rm{N}}_{\rm{i}}}( {{\rm{t}} + {\rm{dt}}} ) = {{\rm{N}}_{\rm{i}}}( {\rm{t}} ){{\rm{e}}^{( {{{\rm{f}}_{\rm{i}}} - \langle {\rm{f}}\rangle } ){\rm{dt}}}}$. At the end of each epoch, we recalculated the mean fitness of the simulated population and reallocated strains between the low and high population-size groups. The threshold population size at which a strain is reallocated (${\epsilon _{\rm{i}}}$) is strain specific and given by ${\epsilon _{\rm{i}}}{\rm{ = \ }}\frac{{\rm{\xi }}}{{{{\rm{f}}_{\rm{i}}}{\rm{ - }}\langle {\rm{f}}\rangle }}$, where ${\rm{\xi }}$ is a constant factor (we chose ${\rm{\xi \ }} = {\rm{\ }}3$).

We modeled the generation of new strains due to mutation using a two-step process. We first draw the number of mutants each strain will generate in a given epoch from a Poisson distribution with an expectation value for each strain ${{\rm{\mu }}_{\rm{i}}} = {{\rm{N}}_{\rm{i}}}\ {{\rm{U}}_{\rm{b}}}{\phi _{\rm{i}}}{\rm{dt}}$, where ${{\rm{N}}_{\rm{i}}}$ is the strain's population size, ${{\rm{U}}_{\rm{b}}}$ is the per genome per generation beneficial mutation rate (set at ${10^{ - 5.5}}\frac{{{\rm mutations}}}{{{\rm{genome\ - \ gen}}}}$), and ${\rm{dt}}$ is the epoch duration. ${\phi _{\rm{i}}}$ is calculated as the fraction of missense mutations in a genetic code that do not result in truncation, normalized by that same fraction for the Standard Code. We ignore deleterious and neutral mutations because, under strong selective pressure, the evolution of a population is largely determined by beneficial mutations (44).

Each mutation is then assigned a fitness effect (${\rm{d}}{{\rm{f}}_{\rm{i}}}$) drawn from a Distribution of Fitness Effects (DFE). We modeled the DFE with a generalized half-normal distribution (${\rm{P\ }}( {{\rm{df}}} ) = {\rm{\ }}\frac{{{\rm{\beta \lambda }}}}{{2{\rm{\ \Gamma }}( {\frac{1}{{\rm{\beta }}}} )}}{\rm{\ }}{{\rm{e}}^{ - {{( {{\rm{\lambda \ df}}} )}^{\rm{\beta }}}}}$). Parameters were set at ${\rm{\beta \ }} = \ 1$ and ${\rm{\lambda \ }} = \ 2$ such that the average mutation would have a fitness effect equal in magnitude to that empirically determined in (45). Stated differently, we randomly assigned the strength of each mutation such that the average mutation was weakly beneficial (i.e. ∼2% faster doubling than the parental strain) and that stronger mutations were exponentially less likely to occur. We then introduce a new strain for each mutation with population size ${{\rm{N}}_{\rm{i}}} = {\rm{\ }}1$ and fitness ${{\rm{f}}_{\rm{i}}} = {{\rm{f}}_{\rm{j}}}{\rm{\ }} + {\rm{d}}{{\rm{f}}_{\rm{i}}}$, where ${{\rm{f}}_{\rm{j}}}$ is the fitness of the parent strain from which the new strain mutated.

We also used two approximations to reduce computational costs. Theory suggests that mutants generated from strains with low population-sizes have a vanishingly low probability of establishing in the population (44). Thus in our first approximation, we did not generate mutants originating from the small population-size group. Our second approximation prematurely removed low fitness strains from the population once two conditions are met: (i) the mean fitness of the population surpassed the fitness of that strain and (ii) the population size of that strain is low enough to move the strain to the small population-size group. Our second assumption artificially inflates the mean fitness of the simulated population by ∼0.03%.

### Preparing expression plasmids

We received pSB1C3-T7-sfGFP from Eric Wei for use as the standard-encoded expression vector (sfGFP_SC) as well as the backbone for our RED20-encoded expression vector (sfGFP_RED20). To produce sfGFP_RED20, we computationally recoded the coding sequence of super-folder green fluorescent protein (sfGFP) to only include codons used by RED20. The recoded gene was then synthesized by Integrated DNA Technologies (IDT) as a gBlock and assembled into pSB1C3-T7 using the NEB HiFi Assembly kit (NEB# E5520S) to produce sfGFP_RED20.

Chemically competent *Escherichia coli* Top10 cells were incubated with 2.5 μl of assembly product on ice for 30 min. These cells were then heat shocked at 42°C for 30 s, returned to ice for 2 min, and grown out in 950 μl SOC media at 37°C for 1 h. The resulting transformants were plated on LB agar with chloramphenicol (25 ng/μl) and grown over night at 37°C with shaking. Colonies were then grown up in 50 ml TB broth with chloramphenicol (25 ng/μl) for 16 h at 37°C with shaking. Each overnight culture was split into five batches of 10 ml each, and plasmid was prepared from each batch separately using QIAprep Spin Miniprep kits (QIAGEN, Cat No./ID: 27104) and then pooled. Final DNA product was assessed for quantity and purity using a NanoDrop 2000 (Thermo Scientific). Annotated sequence maps for sfGFP_SC (https://benchling.com/s/seq-gqXNUQJ41NbxOmdFD3LN) and for sfGFP_RED20 (https://benchling.com/s/seq-w63RBxrXRxi6uIruvKEM) are freely available.

### Expressing protein *in vivo*

Chemically competent *E. coli* BL21(DE3) cells were transformed with either sfGFP_SC or sfGFP_RED20 as described above. Transformants were then plated and individual colonies were grown up in 5 ml LB broth with chloramphenicol (25 ng/μl) for 12 h at 37°C with shaking. These cultures were then back diluted in fresh media to an OD600 of 0.5, induced with Isopropyl β-d-1-thiogalactopyranoside (IPTG) to a final concentration of 1 mM, and incubated at 37°C with shaking. After induction, cells were photographed under blue light.

### Expressing protein and measuring fluorescence *in vitro*

We identified 20 elongator tRNA species, one for each conventional amino acid, as well as the initiator tRNA from *E. coli* (Supplementary Table S1). In cases where *E. coli* encodes multiple tRNA species decoding the same codon (e.g. tRNA^Thr^, tRNA^Tyr^ and tRNA^Val^) we chose specific tRNA whose biochemical function had been previously assayed *in vitro*, as possible (46–49). The resulting 21 tRNA sequences were obtained individually by direct RNA synthesis without any base modifications (Agilent Technologies) and resuspended in nuclease free TE buffer at pH 8.0. Individual tRNAs were combined in equimolar ratio at 250 μM each to create a RED20 tRNA 25× master mix (10 μM final concentration per tRNA). An *in vitro* RED20 prototype was prepared by supplementing a PURExpress *in vitro* expression system lacking tRNAs (PURE ΔtRNA, NEB# E6840S). A standard-encoded expression system was built by supplementing PURE ΔtRNA with control tRNAs supplied by NEB. We added 1 μl of murine RNase inhibitor to all *in vitro* reaction (NEB# M0314S). Each reaction also received 60 pmol of either the RED20-encoded or standard-encoded expression vector. Otherwise, reactions were assembled as specified by NEB to a final volume of 10 μl.

Reactions were carried out in a SpectraMax i3 plate reader (Molecular Devices) using clear bottom, 384-well microtiter plates (Corning) at 37°C for 16 h. Protein expression was measured using the same plate reader. Samples were excited at 485 nm (9 nm bandwidth) and emission was measured at 520 nm (15 nm bandwidth) every two minutes following 3 s of shaking.

## RESULTS

### Fail-safe codes lacking translation machinery for a subset of codons are designed to penalize missense mutation

**Figure 2.**
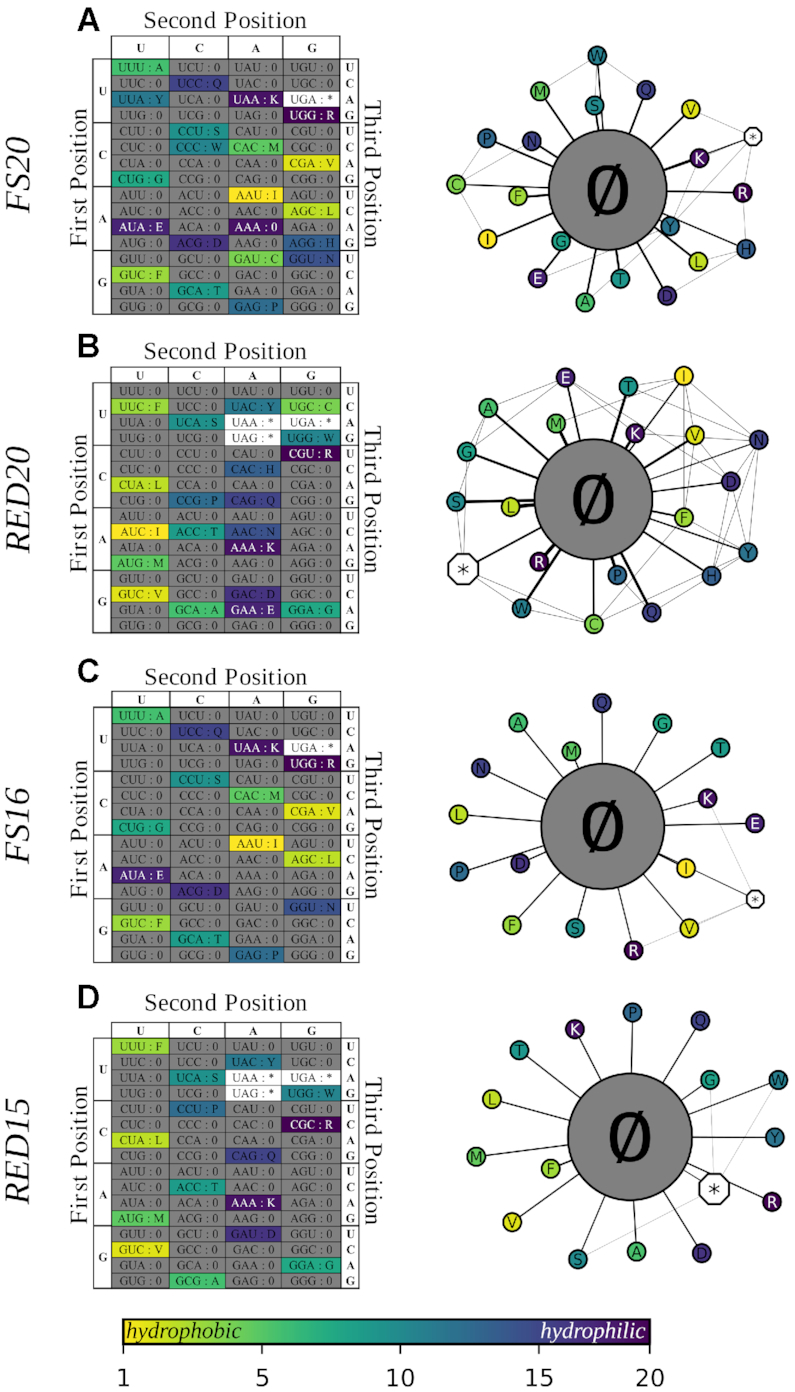
Genetic codes can be designed to map mutations from sense codons to null codons. Table and mutation-distance network representations of fail-safe codes. (**A**) FS20 requires synthetic translation machinery. (**B**) RED20 can be realized using *E. coli* translation machinery. Both FS20 and RED20 support expression with the full set of proteinogenic amino acids. (**C**) FS16 requires synthetic translation machinery. (**D**) RED15 can be realized using *E. coli* translation machinery. Both FS16 and RED15 support expression with a reduced set of amino acids such that all point mutations map to null codons. We omit specific amino acids in accordance with specific rationales (Supplementary Figure S1).

We designed fail-safe genetic codes with reduced sets of translation machinery as necessary to encode each expressible amino acid, eliminating degenerate sense codons as possible (Figure 2). Most codons in our reduced codes are ‘null codons,’ meaning they should not be specifically recognized by any tRNAs or translation factors. Genes designed for such fail-safe codes would be encoded using the single, specific sense codon designated for each amino acid. Mutations in so-encoded open reading frames (ORFs) would most typically result in null codons. Previous work has shown that deletions of tRNAs or release factors that remove all machinery decoding a particular codon are either strongly deleterious or lethal, implying that attempting to translate null codons should slightly reduce organismal fitness (50,51).

As a first example, we designed a family of fail-safe codes that map 20 sense codons uniquely to 20 amino acids and one codon to a stop signal. These codes are each instantiated with 20 synthetic elongator tRNAs, one synthetic initiator tRNA, and one synthetic release factor. We call these codes ‘Fail-Safe 20,’ or FS20, because they support expression of all 20 conventional amino acids. There are ${\rm{P}}( {64,{\rm{\ }}21} ) \approx 2{\rm{\ }} \times {\rm{\ }}{10^{36}}$ unique FS20 codes, one of which optimized to map single point mutations to null codons is shown (Figure 2A). FS20 codes that map the maximal fraction of single point mutations to null codons have the same number of sense codons adjacent only to null codons, and of sense codons adjacent to each other via point mutation. However, the set of sense codons adjacent to each other via point mutation differs for each FS20 code. Engineers might therefore encode engineered organisms using FS20 codes that maximize the likelihood of null codon mutations for any given proteome. While our designs for FS20 codes anticipate eventual advances in synthetic biology sufficient to realize entirely arbitrary genetic codes, building most FS20 codes today would be nontrivial. Specifically, most FS20 codes would require codon reassignments requiring significant tRNA and tRNA synthetase engineering. While codon reassignment has been well explored for use with non-natural amino acids involving a few codons, such reassignment has not been reported for all 64 codons (52–58).

To avoid reengineering all tRNAs and tRNA synthetases, we next considered synthetic genetic codes that reuse the translation machinery already implementing the Standard Code. Such genetic codes can be readily realized by reusing naturally occurring molecules. As a first example, we designed a ‘reduced’ fail-safe code we named RED20 that maps 20 sense codons uniquely to 20 amino acids, and three codons to stop signals (Figure 2B). RED20 would require 20 natural elongator tRNAs, one natural initiator tRNA, and the set of natural release factors. RED20 is one instance of a family of $ \approx 3{\rm{\ }} \times {\rm{\ }}{10^8}$ similar codes and, like FS20, is optimized to map single point mutations to null codons, thereby increasing the fraction of deleterious or lethal mutations. Practically, RED20 can be instantiated by natural tRNAs sourced from *E. coli* (Supplementary Table S1).

### Fail-safe codes with reduced amino acid sets or quadruplet codons only map mutations to null codons

While FS20 and RED20 are designed to maximize the fraction of coding-sequence mutations mapping to null codons and minimize the fraction of missense mutations, it is impossible to encode 20 amino acids in a 64-codon genetic code such that each sense codon is only immediately adjacent to null codons. To ensure that all mutations from sense codons map to null codons, we considered either encoding fewer amino acids or adopting a larger codon table.

We designed a family of fail-safe codes based on the FS20 codes that only encode reduced sets of 16 amino acids (hereafter FS16, Figure 2C). FS16 codes map 16 sense codons uniquely to 16 amino acids and one codon to a stop signal. FS16 codes are each instantiated with 16 synthetic elongator tRNAs, one synthetic initiator tRNA, and one synthetic release factor. There are ${\rm{\ P}}( {64,{\rm{\ }}17} ){\rm{\ }} \times {\rm{\ P}}( {20,{\rm{\ }}16} ) \approx 5{\rm{\ }} \times {\rm{\ }}{10^{46}}$ unique FS16 codes, one of which designed to map all single point-mutations to null codons is shown (Figure 2C). Similarly, we designed a fail-safe code based on RED20 that maps 15 sense codons uniquely to 15 amino acids and three codons to stop signals (hereafter RED15, Figure 2D). RED15 is instantiated with 15 natural elongator tRNAs, one natural initiator tRNA, and the set of natural release factors. RED15 is a member of a family of $ \approx 2{\rm{\ }} \times {\rm{\ }}{10^{11}}$ similar codes and, like FS16, is designed to map all single point mutations to null codons. As with RED20, RED15 could be instantiated with tRNA sourced from *E. coli* (Supplementary Table S1). Because FS16 and RED15 map all mutations to null codons, we call them ‘ideal’ fail-safe codes. We selected and recommend specific FS16 and RED15 codes on the basis of our own idiosyncratic design principles (e.g. if one of many similar amino acids is encoded then other similar amino acids become less important; Supplementary Figure S1), but encourage other fail-safe code designers to consider different choices (e.g. histidine over glutamine, or vice versa).

We also considered genetic codes with expanded codon sets. Quadruplet decoding occurs in nature (59–61) and has been demonstrated experimentally (55,62–64). While the use of quadruplet codons is currently limited to a few positions per gene (55,63,64), we considered quadruplet codon designs in anticipation of ongoing advances in synthetic biology. Specifically, we designed a family of quadruplet-codon fail-safe codes (hereafter FSQUAD) with 256 available codons (Supplementary Figure S2). FSQUAD codes would be able to encode more than 20 amino acids such that all mutations from sense codons map to null codons, allowing for programmable incorporation of non-natural amino acids in a fail-safe encoded system. Like FS20- or FS16-encoded organisms, an FSQUAD-encoded organism should also be resistant to horizontal gene transfer. Additionally, previous work suggests that quadruplet decoding is adaptive at higher temperatures, which implies that FSQUAD-encoded organisms may be well suited to high-temperature applications (61).

### Simulations quantify relative evolutionary rates of different genetic codes

We simulated large asexual populations of organisms encoded via different fail-safe genetic codes to explore how fail-safe genetic codes might impact behavior over many generations. We developed a hybrid model where small population-size lineages are treated stochastically using a birth-death process to capture genetic drift, and large population-size lineages are treated deterministically with exponential growth. Mutations are generated stochastically, the number of which is dependent on the population size and genetic code used (44,45). The fitness effect of each mutation is drawn randomly from a Distribution of Fitness Effects (DFE). The parameters of the DFE were chosen such that its mean matched the empirically determined average fitness-effect of a mutation (45). Stated differently, we randomly generate the number of mutations, then randomly generate the effect each mutation has on organismal fitness, such that the number and strength of those mutations match what we would expect to observe in an experiment (44).

**Figure 3.**
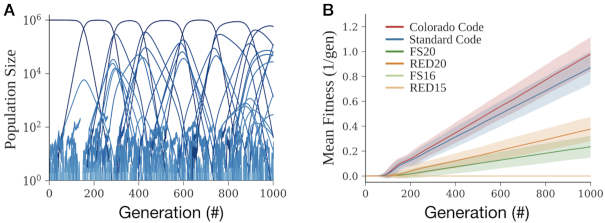
Simulations suggest fail-safe codes should attenuate evolution more effectively than hyperevolvable codes might accelerate evolution. (**A**) A simulation of mutation-selection balance in large, asexual populations. Each line represents the population size of an isogenic lineage versus time. New lineages arise as mutants are generated. (**B**) Mean fitness traces for replicates of populations (*n* = 1000) using the Standard Code (blue), Colorado Code (red), FS20 (dark green), FS16 (light green), RED20 (dark orange), and RED15 (light orange). Mean fitness of a simulated batch culture (bold) and standard deviation across replicates (shaded region) are shown.

During the course of a simulation, an initially monoclonal population generates diversity via mutation. Newer, more fit strains arise and slowly outcompete less fit strains, increasing the mean fitness of the population (Figure 3A). We compare evolutionary rates of genetic codes by comparing the rates at which the mean fitnesses of populations encoded in these codes change over time (Figure 3B). For example, for our chosen parameters, we predict the Standard Code allows fitness to increase at a rate of $8.71{\rm{\ }} \times {\rm{\ }}{10^{ - 4}} \ 1/{\rm{ge}}{{\rm{n}}^2}{\rm{\ }}$(s.d $1.31{\rm{\ }} \times {\rm{\ }}{10^{ - 4}} \ 1/{\rm{ge}}{{\rm{n}}^2}$). With the same parameters, the Colorado Code is expected to evolve only 12% faster ($9.79{\rm{\ }} \times {\rm{\ }}{10^{ - 4}} \ 1/{\rm{ge}}{{\rm{n}}^2}$, s.d. $1.36{\rm{\ }} \times {\rm{\ }}{10^{ - 4}} \ 1/{\rm{ge}}{{\rm{n}}^2}$).

The fail-safe codes studied here are expected to have a much stronger effect on evolutionary dynamics (Table 1). For example, FS20 reduced predicted evolutionary rates by 73% compared to the Standard Code ($2.35{\rm{\ }} \times {\rm{\ }}{10^{ - 4}} \ 1/{\rm{ge}}{{\rm{n}}^2}$, s.d. $0.877{\rm{\ }} \times {\rm{\ }}{10^{ - 4}} \ 1/{\rm{ge}}{{\rm{n}}^2}$). RED20 behaves qualitatively similarly to FS20, despite its imposed design constraints, yielding an expected evolutionary rate only 43% that of the Standard Code ($3.77{\rm{\ }} \times {\rm{\ }}{10^{ - 4}} \ 1/{\rm{ge}}{{\rm{n}}^2}$, s.d. $0.977{\rm{\ }} \times {\rm{\ }}{10^{ - 4}} \ 1/{\rm{ge}}{{\rm{n}}^2}$). The ideal fail-safe codes FS16 and RED15 were predicted to arrest ORF evolution due to single point mutations altogether.

**Table 1.** Evolutionary rates are expected to vary across natural and fail-safe genetic codes. Predicted evolutionary rate is reported as the change in fitness (in units of $1 / {\rm{gen}}$) per unit time (in units of ${\rm{gen}}$). Mean rate of fitness increase is reported along with standard deviation. Codes marked with an asterisk were simulated both with and without considering tRNA promiscuity

Genetic code	Evolutionary rate ($1/{\rm{ge}}{{\rm{n}}^2}$)
Standard Code	$8.7\ \times {10^{ - 4}} \pm 1.3\ \times {10^{ - 4}}$
Colorado	$9.8\ \times {10^{ - 4}} \pm 1.4\ \times {10^{ - 4}}$
FS20	$2.4\ \times {10^{ - 4}} \pm 0.9\ \times {10^{ - 4}}$
FS16	$0.0\ \times {10^{ - 4}} \pm 0.0{\rm{\ }} \times {10^{ - 4}}$
RED20*	$3.8\ \times {10^{ - 4}} \pm 0.4\ \times {10^{ - 4}}$ (no wobbling)
	$5.9\ \times {10^{ - 4}} \pm 1.4\ \times {10^{ - 4}}$ (with wobbling)
RED15*	$0.0\ \times {10^{ - 4}} \pm 0.0\ \times {10^{ - 4}}$ (no wobbling)
	$3.2\ \times {10^{ - 4}} \pm 0.9\ \times {10^{ - 4}}$ (with wobbling)
FSQUAD	$0.0\ \times {10^{ - 4}} \pm 0.0\ \times {10^{ - 4}}$

### Biocontainment may arise intrinsically in organisms using fail-safe genetic codes

**Figure 4.**
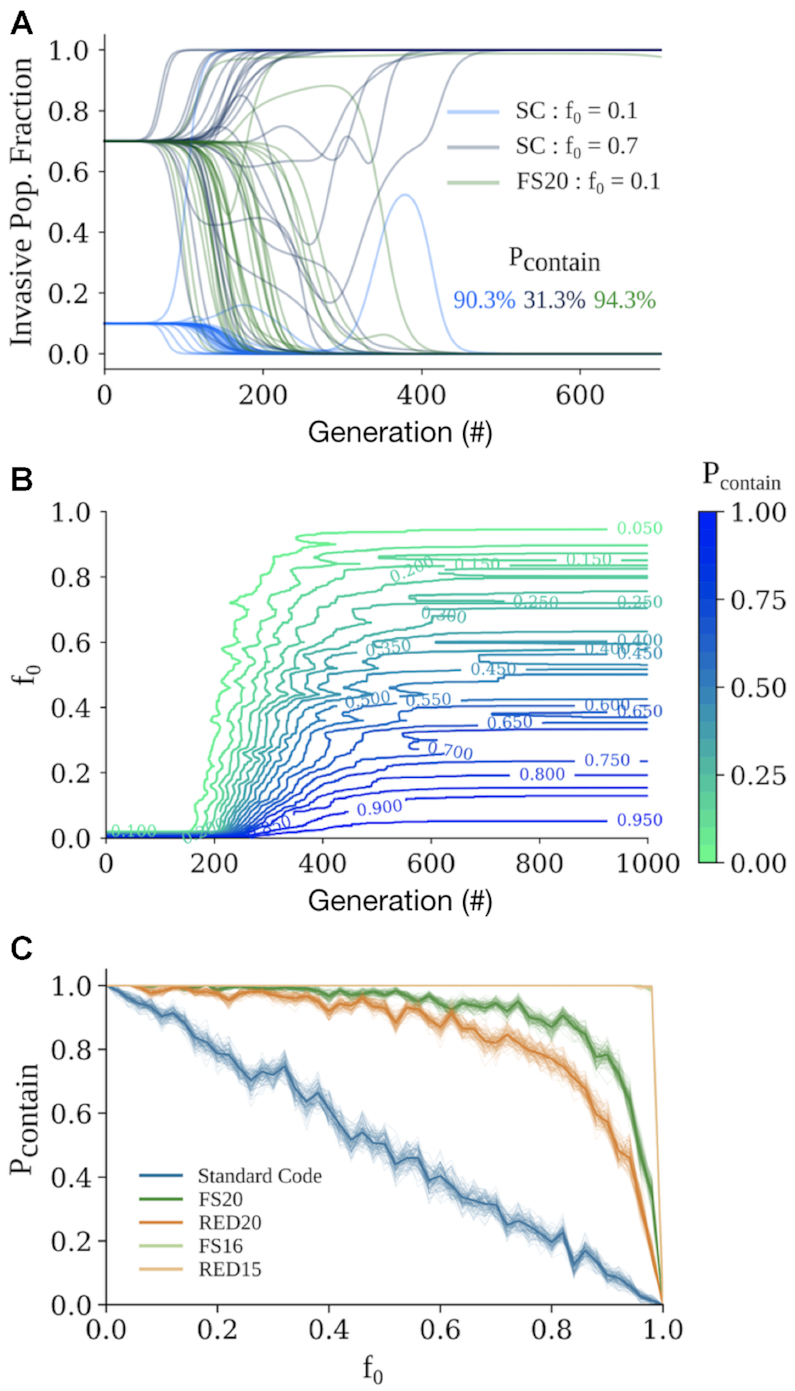
Fail-safe codes may also prevent organisms from escaping into the environment. (**A**) Replicates (*n* = 300) of simulated competition between a native population encoded in the Standard Code and a monoclonal invasive population either encoded in the Standard Code with an initial population fraction ${{\rm{f}}_0}$ = 10% (light blue) or 70% (dark blue), or encoded in FS20 with ${{\rm{f}}_0}$ = 70% (green). We approximate containment probability ${{\rm{P}}_{{\rm{contain}}}}$ as the fraction of simulations in which the invasive population is eliminated. (**B**) Contour graphs of containment probability vs. time (x axis) and ${{\rm{f}}_0}$ (y axis) for invasive strains using the Standard Code (*n* = 300 replicates). Color represents ${{\rm{P}}_{{\rm{contain}}}}$ magnitude, varying from 0 (green) to 1 (blue). ${{\rm{P}}_{{\rm{contain}}}}$ reaches a steady state value at the limit of large $\rm{t}$. (**C**) ${{\rm{P}}_{{\rm{contain}}}}$ at steady state versus ${{\rm{f}}_0}$ for invasive strains using fail-safe codes (*n* = 300 replicates). Bootstrapped-resampled traces of the data (lighter shaded lines) are shown with colors as in Figure 3B.

We hypothesized that fail-safe encoded organisms will adapt to new environments more slowly than naturally-encoded organisms and thus might be less able to displace established populations. If true, then fail-safe encoding could be used as an intrinsic biocontainment layer, one that does not rely on a heterologous genetic function but rather is instantiated via the encoding of the entire organism. To quantitatively assess this possibility, we simulated competing populations of organisms encoded by both standard and fail-safe codes, exploring when and to what extent invading populations might displace established populations. In our simulations, the invasive populations either swept or were swept by the native populations (Figure 4A). More specifically, we defined a containment probability, ${{\rm{P}}_{{\rm{contain}}}}( {{{\rm{f}}_0},{\rm{\ t}},{\rm{\ }}\mathbb{T}} )$, as the likelihood that the invasive population will have been outcompeted by time ${\rm{t}}$, given an initial invasive population fraction ${{\rm{f}}_0}$ and genetic code $\mathbb{T}$. After sufficient time, the containment probability reaches a steady state, varying only in initial population fraction (Figure 4B, Supplementary Figure S5). We estimated the steady state containment probability versus initial invasive population fraction for our fail-safe codes (Figure 4C). We predict FS20 will maintain a containment probability ${{\rm{P}}_{{\rm{contain}}}} < 99{\rm{\% }}$ up to an initial invasive population fraction ${{\rm{f}}_0} \le 36{\rm{\% }}$. RED20 was able to maintain ${{\rm{P}}_{{\rm{contain}}}} < 99{\rm{\% }}$ up to ${{\rm{f}}_0} \le 14{\rm{\% }}$. We predict organisms encoded in FS16 and RED15 would be outcompeted across all initial conditions simulated. Our theoretical results suggest that population-level biocontainment is expected to be an intrinsic property of organisms encoded via fail-safe codes.

**Figure 5.**
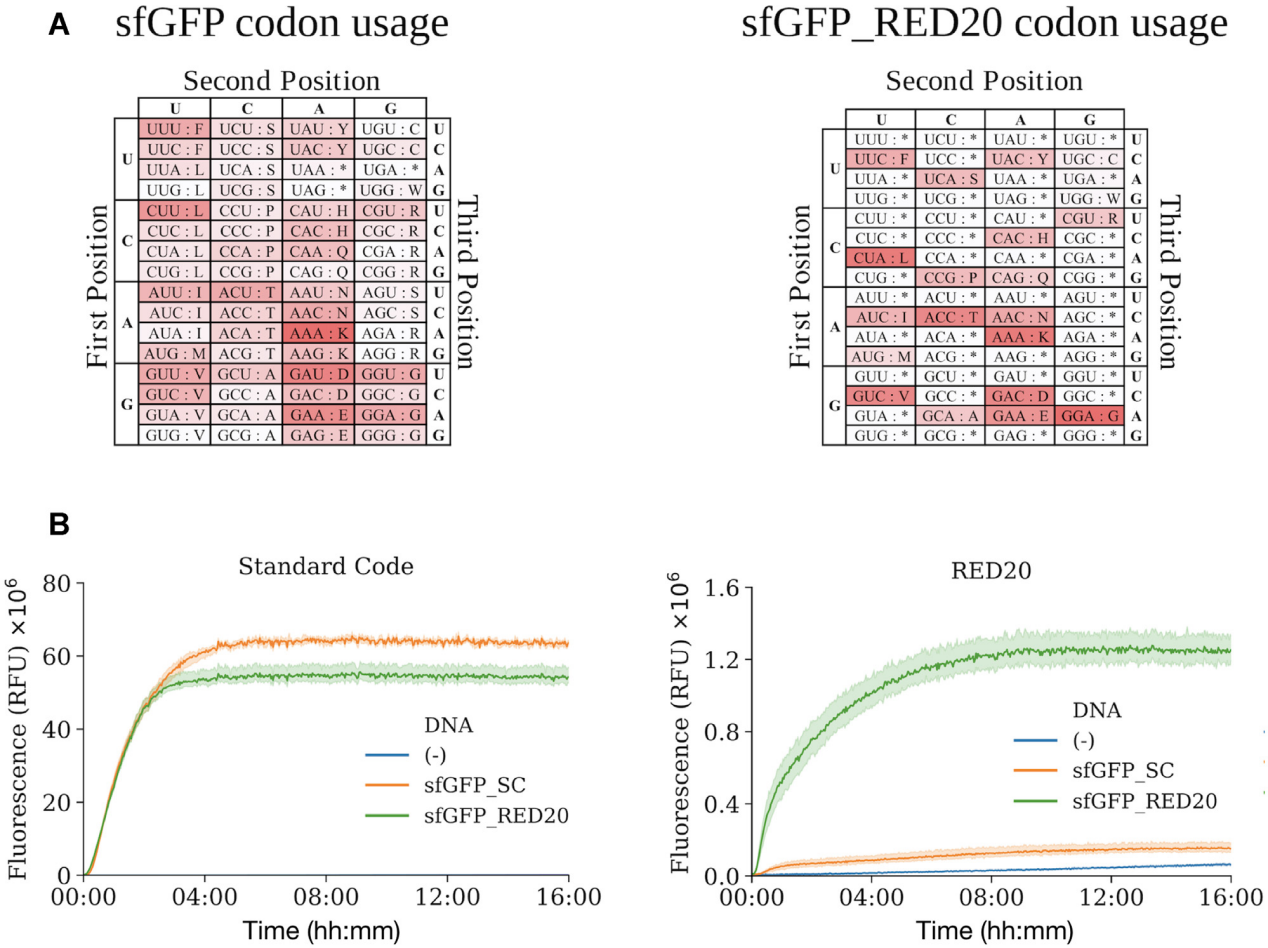
A reduced set of tRNA encoding RED20 can express a functional fluorescent protein. (**A**) Frequency of codon usage in coding sequences for super folder variants of GFP (sfGFP) encoded in either the Standard Code (sfGFP_SC, left) or RED20 (sfGFP_RED20, right). Unused codons are represented in white, while frequently used codons are represented in red. (**B**) Fluorescence versus time for sfGFP encoded in the Standard Code (orange, *n* = 3) or in RED20 (green, *n* = 4) expressed *in vitro* from a tRNA set encoding either the Standard Code (left) or RED20 (right). Reactions without template DNA were included as negative controls (blue, *n* = 3). Mean fluorescence for a given condition, averaged across replicates (bold lines) and standard deviation across replicates (shaded regions) are shown.

### A reduced set of tRNAs instantiating RED20 enables protein expression

We sought to learn if any of our fail-safe codes might actually work. As a first test, we encoded the superfolder green fluorescence protein (sfGFP) in both the Standard Code (sfGFP_SC) and in RED20 (sfGFP_RED20). We transformed plasmids expressing each gene into *E. coli* containing a full complement of natural tRNA. We observed that both encodings of sfGFP were well expressed (Supplementary Figure S6). We next sought to test if a reduced set of tRNA would express the reduced encoding of sfGFP but not the standard encoding. Since a RED20-encoded organism does not yet exist, we created a chemically-defined *in vitro* expression system with a tRNA set instantiating RED20. Specifically, we obtained a variant of PURE—the *in vitro* translation system composed of individually purified components (65)—that was lacking all tRNA (PURE ΔtRNAs). We were unable to source naturally produced tRNAs at high purity, so we elected to obtain chemically synthesized tRNAs. We designed a set of 20 elongator tRNAs plus an initiator tRNA that instantiate RED20 and procured them via commercial direct-RNA synthesis; these synthetic tRNAs lacked all base modifications known to affect tRNA function (42,66–68). We combined PURE ΔtRNAs with the 21 synthetic tRNA to make an *in vitro* RED20 expression system (PURE RED20). We used bulk purified tRNAs to reconstitute standard-code PURE as a control (PURE). We found that PURE RED20 expressed RED20-encoded, but not standard-encoded, fluorescent protein (Figure 5). Specifically, we observed that our RED20 system expressed RED20-encoded sfGFP at a level 8-fold higher than standard-encoded sfGFP (Figure 5B).

## DISCUSSION

We designed fail-safe genetic codes that lack translation machinery recognizing the majority of codons such that individual point mutations in protein coding sequences should be deleterious to the host organism. We simulated the evolution of populations using these codes to predict the expected effects of fail-safe genetic codes on evolutionary dynamics. Our fail-safe codes are predicted to reduce evolutionary rates in protein coding sequences to ∼30% of the standard code while encoding a full set of 20 conventional amino acids, and to select against all individual point mutations in organisms encoding only 15 or 16 amino acids. The most immediately practical codes, RED20 and RED15, should not require any tRNA or tRNA synthetase engineering to implement. As a first test, we built one RED20 code *in vitro* and demonstrated expression of a protein encoded by only 20 sense codons.

### Fail-safe codes may serve as a base layer for biocontainment strategies

Previous work has focused on containing organisms to prescribed physical niches (23–32). However, full control of reproducing populations will also require containing organisms to prescribed genotypes. To ensure the reliability and long-term stability of synthetic genetic programs, we also need genetic containment methods. Our work suggests that fail-safe codes can offer both physical and genetic containment. Specifically, we predict that fail-safe encoded organisms will not only explore genotype space slower than organisms encoded using the standard code, but will also be less likely to outcompete native populations in new environmental contexts. Organisms encoded with fail-safe codes such as FS20 or FS16 would additionally be genetically isolated from natural organisms (56,69). We believe that fail-safe codes could be used as a base containment layer upon which additional safeguards can be added modularly (26).

### Limitations of our evolutionary model highlight future work in designing fail-safe codes

Our computational model of evolving populations is oversimplified compared to the actual complexity of biology. For example, in our model we assume a ‘flat mutation’ rate, meaning that all base substitutions are equally likely to occur. Empirical studies refute this assumption, suggesting separate mutation rates for ‘transitions’ (purine-to-purine or pyrimidine-to-pyrimidine) and ‘transversions’ (purine-to-pyrimidine or pyrimidine-to-purine). We also decided to define our distribution of fitness effects (DFE) independent of the identities of the amino acids substituted. A more complex model may weigh fitness effect by the magnitude of change between substituted amino acids as measured by a physicochemical metric or empirically determined substitution matrix designed to avoid bias towards any given genetic code (70). A computational model that includes such higher-order considerations may enable design of improved fail-safe codes.

Additionally, we only considered point mutations, which affect just the codon where the mutation occurred. However some mutations, such as insertions or deletions, can disrupt the reading frame in which a gene is translated. Shifting the reading frame not only affects the codon in which a mutation occurs, but also all downstream codons. The Standard Code is naturally robust to frame-shift mutations, encoding ‘hidden’ stop codons that terminate off-frame translation (71). More specifically, when frameshifted proteins are translated, the Standard Code minimizes the effect of the frameshift in two ways: (i) by encoding chemically similar amino acids both off- and on-frame (72) and (ii) by using a subset of 20 codons that form a circular code, meaning that translating these codons in any reading frame will eventually recover the originally encoded signal (73,74). We believe that unlike the Standard Code, fail-safe codes will be sensitive to frameshift mutations. Since frameshift mutations affect multiple codons at a given time, and since any given codon is very likely to map to a null codon upon mutation, then most frameshift mutations should result in multiple null codons. We also expect that many fail-safe codes lack circular codes, despite this not being one of our design considerations. While we expect fail-safe codes will penalize frameshift mutations, future work may wish to further optimize fail-safe codes by explicitly considering frameshifting.

### RED20 *in vitro* expression has a low signal and a high noise floor

The RED20 encoding of green fluorescent protein expressed ∼50-fold less well in a cell-free expression system containing only 21 synthetic tRNA compared to expression via a full set of natural tRNA sourced directly from cells. We believe this difference is due to either a decrease in the total protein-expression capacity of our RED20 system, or to a reduced fraction of functional protein relative to total protein produced. More specifically, since tRNA base modifications are known to affect tRNA function during translation, it is possible that our unmodified synthetic tRNAs may reduce the efficiency of our translation system and thus reduce the total protein produced (42,66–68). It is also possible that our unmodified tRNAs have a reduced codon specificity, causing an increased misincorporation rate and thus a decreased total fraction of correctly-expressed protein. Future work could quantify the amount and identity of translated products from an *in vitro* RED20 expression system to address this question. Alternatively, optimizing buffer composition or tRNA concentrations may reduce this difference.

We also observed that our RED20 expression system made small amounts of fluorescent protein from a standard encoding of the gene, which theoretically should not be expressed at all by RED20. We believe the flourescence signal is above the experimental noise floor as set by a total absence of template DNA (Figure 5 and Supplementary Figure S7). We also believe that this signal is not due to residual tRNA in PURE ΔtRNA, given that the observed fluorescence is ∼10 greater than the PURE ΔtRNA control (Supplementary Figure S7). Rather, RED20 may produce functional standard-encoded sfGFP due to promiscuous decoding of null codons. As mentioned, unmodified tRNAs can have reduced codon specificities, which may allow some null codons to be translated at low levels. Future work using appropriately base-modified tRNAs are warranted.

### Wobble decoding presents a general challenge for code engineering

One challenge in code engineering is the tendency for tRNAs to recognize more than one codon due to wobble decoding (43,67,75). For example, designs for a hyperevolvable code generally maximize the diversity of encoded amino acids adjacent to any given sense codon, which can result in an ambiguous code where many codons are recognized by two differentially aminoacylated tRNAs (Supplementary Figure S3). The effect of wobble decoding on fail-safe codes should be less drastic. For example, we simulated the behavior of RED20- and RED15-encoded organisms assuming that tRNAs that could perform wobble decoding as well as cognate decoding (Supplementary Figure S4). Under these assumptions, RED20 and RED15 maintain predicted evolutionary rates 67% and 37% that of the standard code, respectively. We also predict that organisms using RED20 and RED15 maintain a containment probability >95% up to an invading population fraction (${{\rm{f}}_0}$) of 22% and 54%, respectively. Therefore, while engineering one-to-one decoding would improve fail-safe codes, we predict that RED15 and RED20 are robust to wobble decoding even if instantiated using native or near-native tRNA.

Predicting how wobble decoding might affect a quadruplet code is difficult. We might naively assume that the additional base pair in the codon-anticodon complex would allow FSQUAD to encode four times as many amino acids unambiguously. If so, an ideal quadruplet fail-safe code may be able to encode up to 32 sense positions adjacent only to null codons without requiring tRNAs capable of one-to-one decoding. However, engineering a full set of quadruplet-decoding tRNAs, the cognate aminoacyl transferases and translation factors, and maintaining perfect codon–anticodon specificity has not yet been demonstrated.

### Reduced amino acid sets may still encode interesting biological functions

One way to increase the probability of mutating to a null codon in a fail-safe code is to decrease the number of encoded amino acids, thereby decreasing the number of required sense codons. But what biological functions can be encoded with fewer than twenty amino acids? Could a whole organism ever be encoded with a reduced amino acid set? Of the twenty proteinogenic amino acids, ten are predicted to have resulted from biosynthesis in early terrestrial organisms (76–78). This implies relevant biological functions and perhaps entire organisms may have been encoded with as few as ten amino acids.

As one starting point, Akanuma, Kigawa, and Yokoyoma demonstrated a functional 213 residue enzyme depleted entirely of seven amino acids, including four of the five amino acids we removed in RED15 (79). As a second step towards a reduced amino acid set organism, we recently replaced cysteine from all enzymes in the cysteine biosynthesis pathway (78). Additionally, via a search the UniProt database (80) we found the antimicrobial peptide acanthoscurrin-2 is naturally encoded only via amino acids in our RED15 code (81). Finally, we predict that a functional GFP should be possible via a RED15 code (Supplementary Table S2 and Supplementary Figure S8). Taken together, while significant additional work would be required to remove four or five amino acids from any known natural organism, as would be needed to realize a FS16 or RED15 code, we believe that reduced amino acid set organisms are possible and should be pursued systematically.

### Gene duplication and tRNA evolution should be expected failure modes

We expect that increasing the rate of mutations to null codons will add a selective pressure for noncognate translation machinery to recognize null codons. For example, ribosomal ambiguity mutations (*ram*) impair the proofreading ability of the ribosome (82,83), increasing the likelihood that a noncognate tRNA can recognize a null codon. While several *ram* mutations have been discovered in ribosomal proteins (84–88) we expect that ram mutations in rRNA (89–95) would be more likely to accumulate in any fail-safe encoded organism as designed herein.

We also note that fail-safe codes would not prevent gene duplication. Chromosomal and whole-genome duplication events can result in novel genetic functions (96–98), frequently as a response to stress (99) or other selective pressures (100). Duplication of tRNA genes specifically and subsequent mutation of the anticodon loop has been suggested as a mechanism for genetic code reprogramming in nature (101,102). Such a mechanism could generate tRNAs that recognize null codons, subverting any evolutionary containment strategy based on a fail-safe code. Such failure modes, and likely others, would need to be accounted for and addressed to realize fully non-evolving organisms.

### Removing sense codons from a genome presents a technical challenge

Building a fail-safe encoded organism will require the ability to encode an entire genome such that each amino acid is represented by only one codon. However, codon usage has been shown to regulate gene expression, translation speed, co-translational folding of proteins (103–105), and also the overall fitness of the organism (36). As a result, some synonymous codon substitutions appear disallowed *in vivo* (106). It is an open question how many sense codons are required to instantiate a living organism. Recently, Fredens and colleagues created a synthetic variant of the *E. coli* genome using only 61 codons, 59 of which encode amino acids via synonymous recoding of 18,214 codons plus deletion of otherwise-essential tRNA (107). Additionally, Ostrov and colleagues are working to remove seven sense codons from *E. coli*, creating a 57-codon organism, and have reported successfully recoding 60% of *E. coli* genes (108). While both examples demonstrate genome-scale codon reduction, realizing a 15 or 20 sense-codon organism would require significantly greater genome-scale recoding and codon reduction. We note that recently developed tools for accelerating total genome synthesis have enabled researchers to more rapidly screen recoding strategies, accelerating the pace of progress in the field of codon reduction (106). As engineering whole genomes becomes ever more feasible, so too should designing and building genomes with fewer and fewer sense codons.

## CONCLUSION

We believe that fail-safe codes will play a foundational role in controlling the evolution of biological systems, especially in the context of whole genome engineering. We note several challenges that need to be addressed before any fail-safe organism can be realized. Practically, a subset of our proposed codes do not require reassigning sense codons and instead rely only on the removal of some isoacceptor tRNAs from natural translation systems, greatly simplifying initial experiments. We nevertheless recognize that the proposed work extrapolates far beyond what is currently known. However, given the importance of exploring and realizing non-evolving biological systems we hope that additional academic work on fail-safe codes will be quickly complemented by coordinated professional efforts to realize fail-safe genetic codes and chassis organisms. We believe such work will result in a sort of ‘best available technology’ for realizing responsibly engineered organisms suitable for deployment in field, plant, animal, or patient.

## DATA AVAILABILITY

All code used herein is free online via https://github.com/EndyLab/codon-tables/tree/manuscript. Annotated sequence maps for sfGFP_SC (https://benchling.com/s/seq-gqXNUQJ41NbxOmdFD3LN) and for sfGFP_RED20 (https://benchling.com/s/seq-w63RBxrXRxi6uIruvKEM) can be found online.

## Supplementary Material

gkz745_Supplemental_File
