# Correction to ‘Fail-safe genetic codes designed to intrinsically contain engineered organisms’

**DOI:** 10.1093/nar/gkab1285

**Published:** 2021-12-22

**Authors:** Jonathan Calles, Isaac Justice, Detravious Brinkley, Alexa Garcia, Drew Endy

**Affiliations:** Bioengineering Department, Stanford University, Stanford, CA 94305, USA; Bioengineering Department, Stanford University, Stanford, CA 94305, USA; Department of Mathematics and Computer Science, Claflin University, Orangeburg, SC 29115, USA; Bioengineering Department, Stanford University, Stanford, CA 94305, USA; Bioengineering Department, Stanford University, Stanford, CA 94305, USA

In Materials and Methods, under the section ‘Expressing protein and measuring fluorescence *in vitro*’, there is an error in the sentence ‘Each reaction also received 60 pmol of either the RED20-encoded or standard-encoded expression vector’. The authors used only 60 fmol of DNA template per 10 μl PURE reaction.

The authors apologise for this error.

