# Supplementary material for: Fail-safe genetic codes designed to intrinsically contain engineered organisms
Source: Nucleic Acids Res. 2019 Sep 12;47(19):10439–51. doi: 10.1093/nar/gkz745 (PMC6821295; doi:10.1093/nar/gkz745)
Supplement: gkz745_Supplemental_File [file gkz745_supplemental_file.pdf]

SUPPLEMENTARY INFORMATION

Fail-safe genetic codes designed to intrinsically contain engineered organisms

Jonathan Calles[1], Isaac Justice[1], Detravious Brinkley[2], Alexa Garcia[1], and Drew Endy[1,*],

[1] Bioengineering Department, Stanford University, Stanford, CA, 94305, USA
[2] Department of Mathematics and Computer Science, Claflin University, Orangeburg, SC, 29115, USA

* To whom correspondence should be addressed. Email: endy@stanford.edu

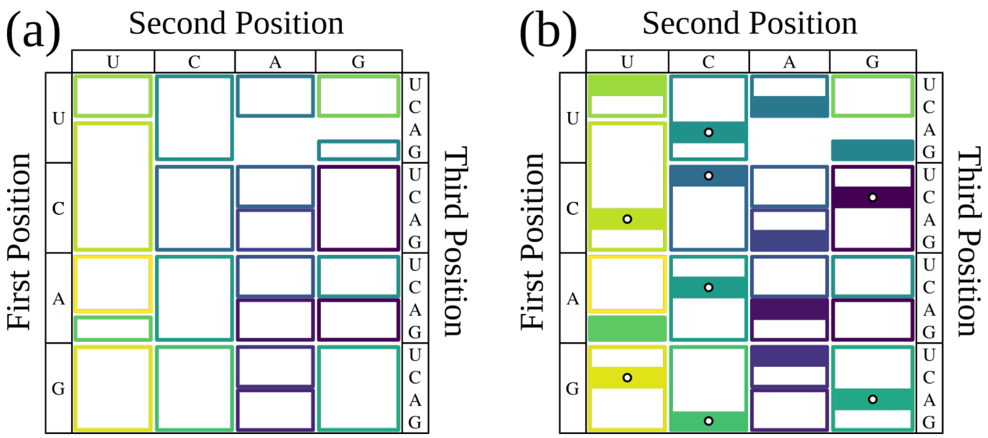

***Supplementary Figure 1: Constructing reduced amino acid set fail-safe genetic codes*.** **(a)** Simplified representation of the Standard Code. Outlined boxes represent blocks of codons that encode the same amino acid. Color represents the rank ordered hydropathy of the encoded amino acid. **(b)** Construction of RED15. Due to the structure of the Standard Code, some pairs of amino acids cannot be simultaneously encoded such that their sense codons are not adjacent by point mutation. These cases occur when two amino acids co-occupy an NNX codon block. For example, aspartate is encoded by GAY and glutamate by GAR, where Y is a pyrimidine (U or C), and R is a purine (A or G). We first assigned amino acids not affected by this constraint (marked by white dots). There are seven amino acid pairs that conflict within an NNX block: phenylalanine (UUY) and leucine (UUR), isoleucine (AUH) and methionine (AUG), histidine (CAY) and glutamine (CAR), asparagine (AAY) and lysine (AAR), cysteine (UGY) and tryptophan (UGG), serine (AGY) and arginine (AGR), and aspartate and glutamate as mentioned previously. In two cases – F and L, and S and R – this conflict can be resolved by encoding one amino acid in another NNX block. As a result, only the following conflicting pairs of amino acids require resolution: I/M, H/Q, N/K, C/W, and D/E. We used

the following biological and biochemical arguments to choose between some pairs of amino acids: M over I to avoid re-engineering translation initiation and due to the physicochemical similarity of isoleucine to leucine; K over N because lysine can form intramolecular salt links and act as a general base; Q over H because glutamine can both accept and donate hydrogen bonds, and to retain one amino acid with an amide side chain; and D over E because aspartate acts as a biosynthetic precursor for methionine, threonine, and lysine [Kanehisa and Goto 2000]. We chose W over C due to the following code structure argument: F (UUY) and Y (AUY) are constrained to two codons each such that either C codon (GUY) would be adjacent by point mutation to F or Y. W (UGG) is not similarly constrained, thus we chose W. We concede convincing arguments can be made for including different combinations of amino acids, but any resulting codes using codons that are nonadjacent by point mutation will have the same predicted evolutionary rate.

	Amino Acid	Locus (*E. coli* K-12 MG1655)	Anti-codon	tRNA Sequence (with Base Modifications)
**RED15**	Phe	chr:3110366-3110441 (+)	GAA	GCCCGGAs4UAGCUCAGDCGGDAGAGCAGGGGAYUGAAms2i6AAYCCCCGUm7Gacp3UCCUUGGm5UYCGAUUCCGAGUCCGGGCACCA
	Leu	chr:696963-697047 (-)	UAG	GCGGGAGUGGCGAAADDGmGUAGACGCACCAGAUUcmo5UAGm1GYUCUGGCGCCGCAAGGUGUGCGAGm5UYCAAGUCUCGCCUCCCGCACCA
	fMet	chr:2947387-2947463 (+)	CAU	CGCGGGGs4UGGAGCAGCCUGGDAGCUCGUCGGGCmUCAUAACCCGAAGm7GUCGUCGGm5UYCAAAUCCGGCCCCCGCAACCA
	Met	chr:696664-696740 (-)	CAU	GGCUACGs4UAGCUCAGDDGmGDDAGAGCACAUCACUac4CAUt6AAYGAUGGGm7Gacp3UCACAGGm5UYCGAAUCCCGUCGUAGCCACCA
	Val	chr:1746435-1746511 (+)	GAC	GCGUUCAs4UAGCUCAGDDGGDDAGAGCACCACCUUGACAUGGUGGGGm7Gacp3UCGUUGGm5UYCGAGUCCAAUUGAACGCACCA
	Ser	chr:1031625-1031712 (-)	UGA	GGAAGUGs4UGGCCGAGCGmGDDGAAGGCACCGGUCmUcmo5UGAms2i6AAACCGGCGACCCGAAAGGGUUCCAGAGm5UYCGAAUCUCUGCGCUUCCGCCA
	Pro	chr:3708616-3708692 (-)	CGG	CGGUGAUUGGCGCAGCCUGGDAGCGCACUUCGUUCGGGm1GACGAAGGGm7GUCGGAGGm5UYCGAAUCCUCUAUCACCGACCA
	Thr	chr:4175754-4175829 (+)	GGU	GCUGAUAUAGCUCAGDDGGDAGAGCGCACCCUUGGUm6t6AAGGGUGAGm7GUCGGCAGm5UYCGAAUCUGCCUAUCAGCACCA
	Ala	chr:3426958-3427033 (-)	UGC	GGGGCUAUAGCUCAGCDGGGAGAGCGCCUGCUUcmo5UGCACGCAGGAGm7GUCUGCGGm5UYCGAUCCCGCAUAGCUCCACCA
	Tyr	chr:4175472-4175556 (+)	GUA	GGUGGGGs4Us4UCCCGAGCGmGCCAAAGGGAGCAGACUQYAms2i6AAYCUGCCGUCACAGACUUCGAAGGm5UYCGAAUCCUUCCCCCACCACCA
	Gln	chr:696430-696504 (-)	CUG	UGGGGUAs4UCGCCAAGCGmGDAAGGCACCGGAUmUCUGm2AYYCCGGCAUUCCGAGGm5UYCGAAUCCUCGUACCCCAGCCA
	Lys	chr:2521253-2521328 (+)	UUU	GGGUCGUUAGCUCAGDDGGDAGAGCAGUUGACUmnm5se2UUUt6AAYCAAUUGm7Gacp3UCGCAGGm5UYCGAAUCCUGCACGACCCACCA
	Asp	chr:228928-229004 (+)	GUC	GGAGCGGs4UAGUUCAGDCGGDDAGAAUACCUGCCUgluQUCm2ACGCAGGGGm7GUCGCGGGm5UYCGAGUCCCGYCCGUUCCGCCA
	Trp	chr:3946957-3947032 (+)	CCA	AGGGGCGs4UAGUUCAADDGGDAGAGCACCGGUCmUCCAms2i6AAACCGGGUm7GUUGGGAGm5UYCGAGUCUCUCCGCCCCUGCCA
	Arg	chr:2817784-2817860 (-)	ACG	GCAUCCGs4UAGCUCAGCDGGADAGAGUACUCGGs2CUICGm2AACCGAGCGm7Gacp3UCGGAGGm5UYCGAAUCCUCCCGGAUGCACCA
	Gly	chr:4175673-4175747 (+)	UCC	GCGGGCAUCGUAUAAUGGCUAUUACCUCAGCCUmnm5UCCAAGCUGAUGAUGCGGGm5UYCGAUUCCCGCUGCCCGCUCCA
**RED20 Only**	Iso	chr:225381-225457 (+)	GAU	AGGCUUGUAGCUCAGGDGGDDAGAGCGCACCCCUGAUt6AAGGGUGAGm7Gacp3UCGGUGGm5UYCAAGUCCACYCAGGCCUACCA
	His	chr:3982510-3982585 (+)	GUG	GGUGGCUAs4UAGCUCAGDDGGDAGAGCCCUGGAUUQUGm2AYYCCAGUUm7GUCGUGGGm5UYCGAAUCCCAUUAGCCACCCCA
	Asn	chr:2044549-2044624 (+)	GUU	UCCUCUGs4UAGUUCAGDCGGDAGAACGGCGGACUQUUt6AAYCCGUAUm7GUCACUGGm5UYCGAGUCCAGUCAGAGGAGCCA
	Glu	chr:2729369-2729444 (-)	UUC	GUCCCCUUCGUCYAGAGGCCCAGGACACCGCCCUmnm5se2UUCm2ACGGCGGUAACAGGGGm5UYCGAAUCCCCUAGGGGACGCCA
	Cys	chr:1991914-1991987 (-)	GCA	GGCGCGUs4UAACAAAGCGGDDAUGUAGCGGAYUGCAms2i6AAYCCGUCUAGUCCGGm5UYCGACUCCGGAACGCGCCUCCA

Supplementary Table 1: Mature Sequences of tRNAs used in RED15 and RED20 genetic codes.

Proposed set of tRNAs for instantiating RED15 and RED20, along with their genomic locations in E. coli (K-12 MG1655) and their mature tRNA sequences with base modifications. Shorthand symbols

for the base modifications used by these tRNA species: D, dihydrouridine; Y, pseudouridine; I, inosine; Q, queuosine; gluQ, glutamyl-queuosine; Um, 2’-o-methyluridine; m5U, 5-methyluridine; s4U, 4-thiouridine; mnm5U, 5-methylaminomethyluridine; mnm5s2U, 5-methylaminomethyl-2-thiouridine; cmo5U, uridine 5-oxyacetic acid; acp3U, 3-(3-amino-3-carboxypropyl)-uridine; Cm , 2’-o-methylcytidine; s2C, 2-thiocytidine; ac4C, N4-acetylcytidine; m2A, 2-methyladenosine; t6A, N6-threonylcarbamoyladenosine; m6t6A, N6-methyl-N6-threonylcarbamoyladenosine; ms2i6A, 2-methylthio-N6-isopentenyladenosine; Gm, 2’-o-methylguanosine; m1G, 1-methylguanosine; m7G, 7-methylguanosine. We require single base changes compared to wild type sequences for 5 tRNA species (proline, alanine, arginine, isoleucine, and cysteine), highlighted in red in each case. Genomic loci and sequences were taken from the GtRNAdb 2.0 database [Chan and Lowe 2016]. Base modifications were taken from the Modomics RNA modification database [Boccaletto et al. 2017].

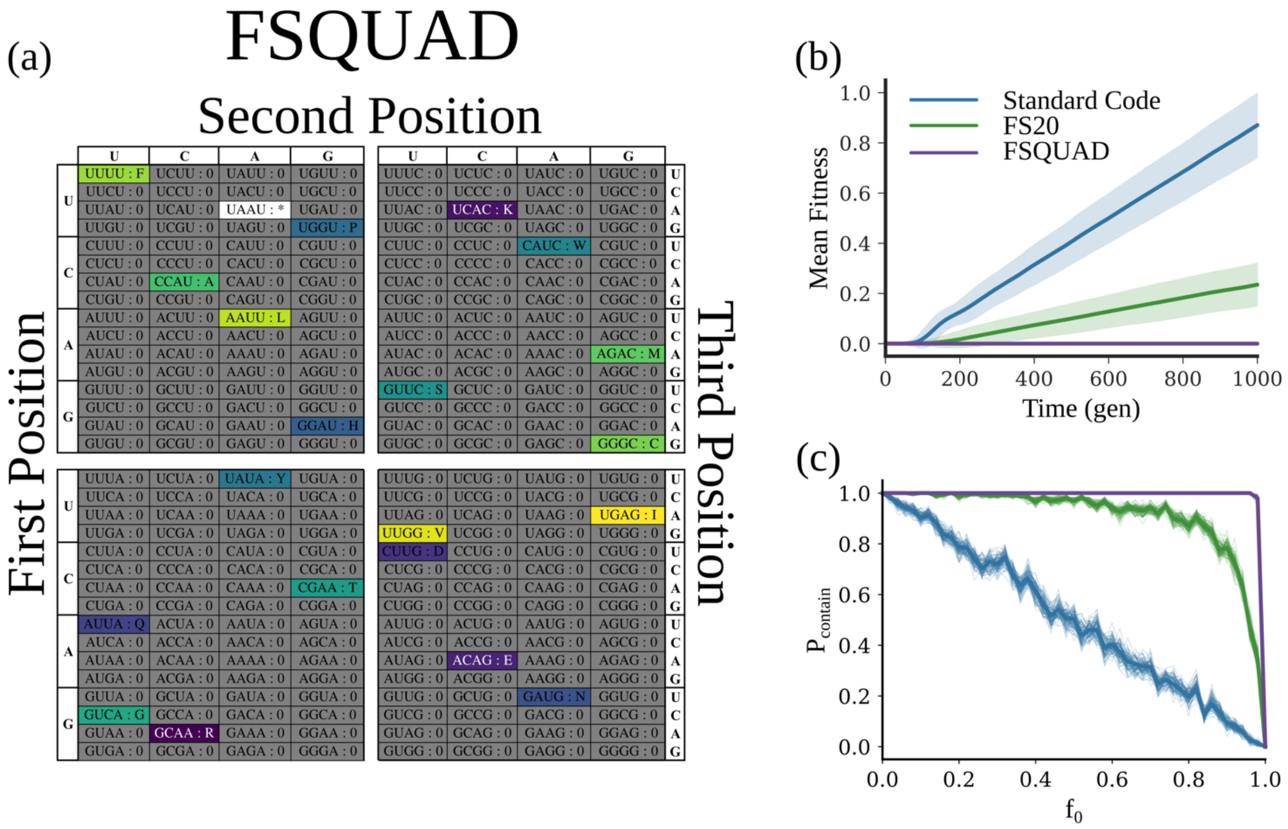

Supplementary Figure 2: Quadruplet fail-safe genetic codes may support expression with 20 or more amino acids without permitting missense mutations. **(a)** Table representation of a quadruplet fail-safe genetic code encoding all 20 proteinogenic amino acids (FSQUAD). Color signifies the rank-ordered hydropathy of the amino acids (I is most hydrophobic, R is most hydrophilic). Quadruplet decoding rather than triplet decoding allows for 256 (4^4) sense codons rather than 64 (4^3). As a result, FSQUAD has a set of 64 codons that all vary from each other at 2 or more positions, rather than the set of 16 available to FS20 or FS16. **(b)** Mean fitness traces for fail-safe codes using triplet and quadruplet-codon decoding (n = 1000 replicates). Bold lines indicate the mean fitness of a batch culture, averaged across replicates. Shaded regions represent the standard deviation of the mean fitness across replicates. The Standard Code is represented in blue. FS20 is

represented in green. FSQUAD is represented in purple. **(c)** $P_{contain}$ at steady state vs. f_0 for invasive strains using fail-safe codes using triplet and quadruplet-codon decoding (n = 300 replicates). Colors are the same as in Fig 6b. Lighter shaded lines represent bootstrap-resampled traces of the data.

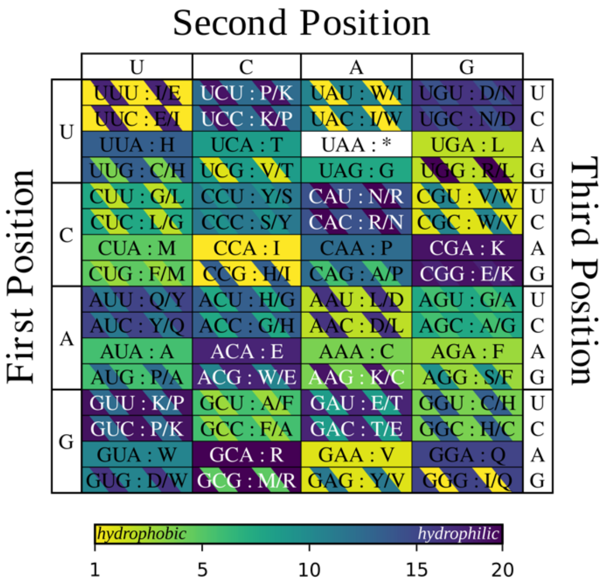

Supplementary Figure 3: Predicted effect of tRNA promiscuity on Colorado Code. The hyperevolvable code proposed by Pines et al. 2017, adjusted to consider ambiguous decoding due to tRNA promiscuity. Color signifies the rank-ordered hydropathy of the amino acids (I is most hydrophobic, R is most hydrophilic). Codons shaded with two colors would be recognized by two tRNA species, with each color corresponding to an amino acid decoded at that codon. For these ambiguous codons the first amino acid listed corresponds to the cognate tRNA, with the second amino acid corresponding to the wobble decoding tRNA (e.g., UUU is assigned to I, but also encodes E by wobble decoding). Only 17 of 64 codons are unambiguously decoded, one of which corresponding to the termination signal.

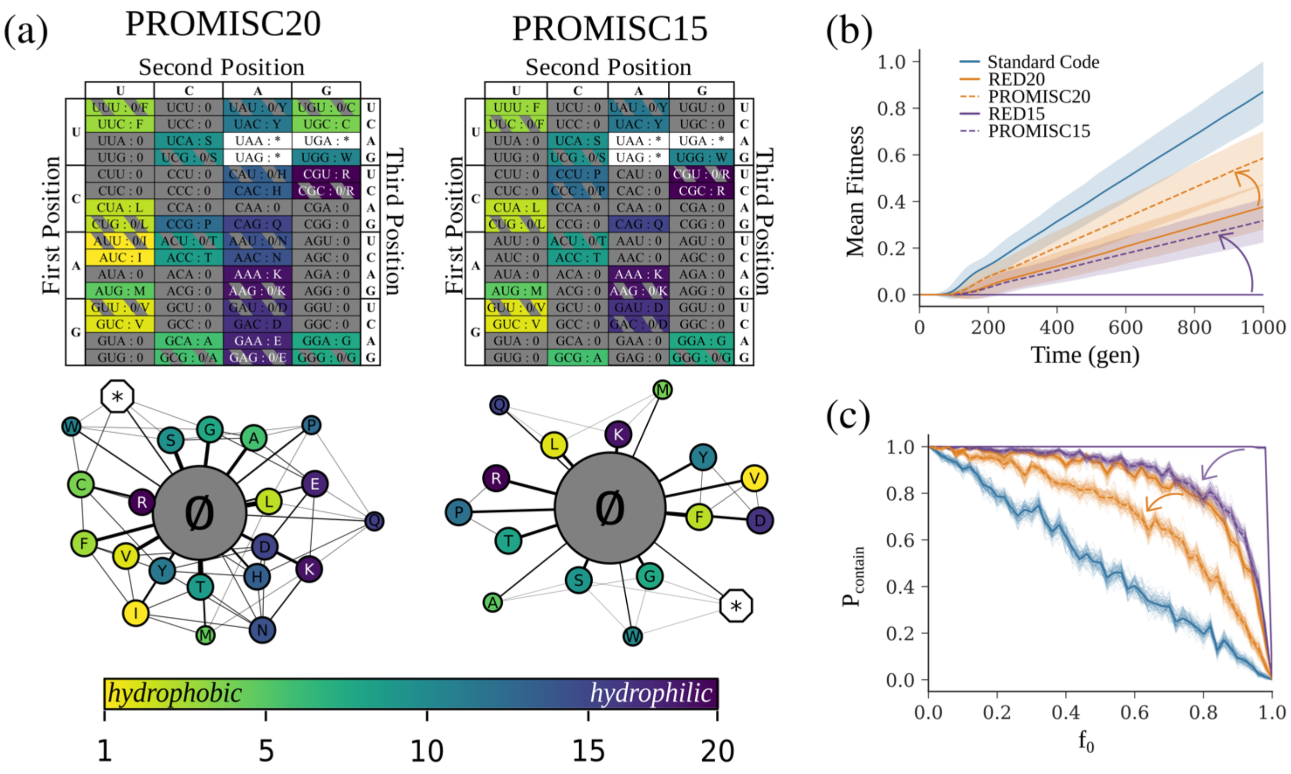

Supplementary Figure 4: Accounting for tRNA promiscuity has a limited effect on the expected evolutionary rates of fail-safe codes. **(a)** Effect of wobble decoding on RED20 and RED15. Table and mutation-distance network representations of the codes that result from considering wobble decoding (PROMISC20 resulting from RED20 and PROMISC15 resulting from RED15). Color signifies the rank-ordered hydropathy of the amino acids (I is most hydrophobic, R is most hydrophilic). Shaded boxes represent null codons that may be recognized by wobble decoding. A detailed discussion of tRNA promiscuous decoding is included in the Materials and Methods section. **(b)** Mean fitness traces for fail-safe codes with and without considering tRNA promiscuity (n = 1000 replicates). Bold lines indicate the mean fitness of a batch culture, averaged across replicates. Shaded regions represent the standard deviation of the mean fitness across replicates. The Standard Code is represented in blue. RED20 and RED15 are represented as the solid and dashed

orange lines respectively. PROMISC20 and PROMISC15 are represented with solid and dashed purple lines respectively. **(c)** $P_{contain}$ at steady state vs. f_0 for invasive strains using fail-safe codes with and without considering wobble decoding (n = 300 replicates). Colors are the same as in Fig. 5b. Lighter shaded lines represent bootstrap-resampled traces of the data.

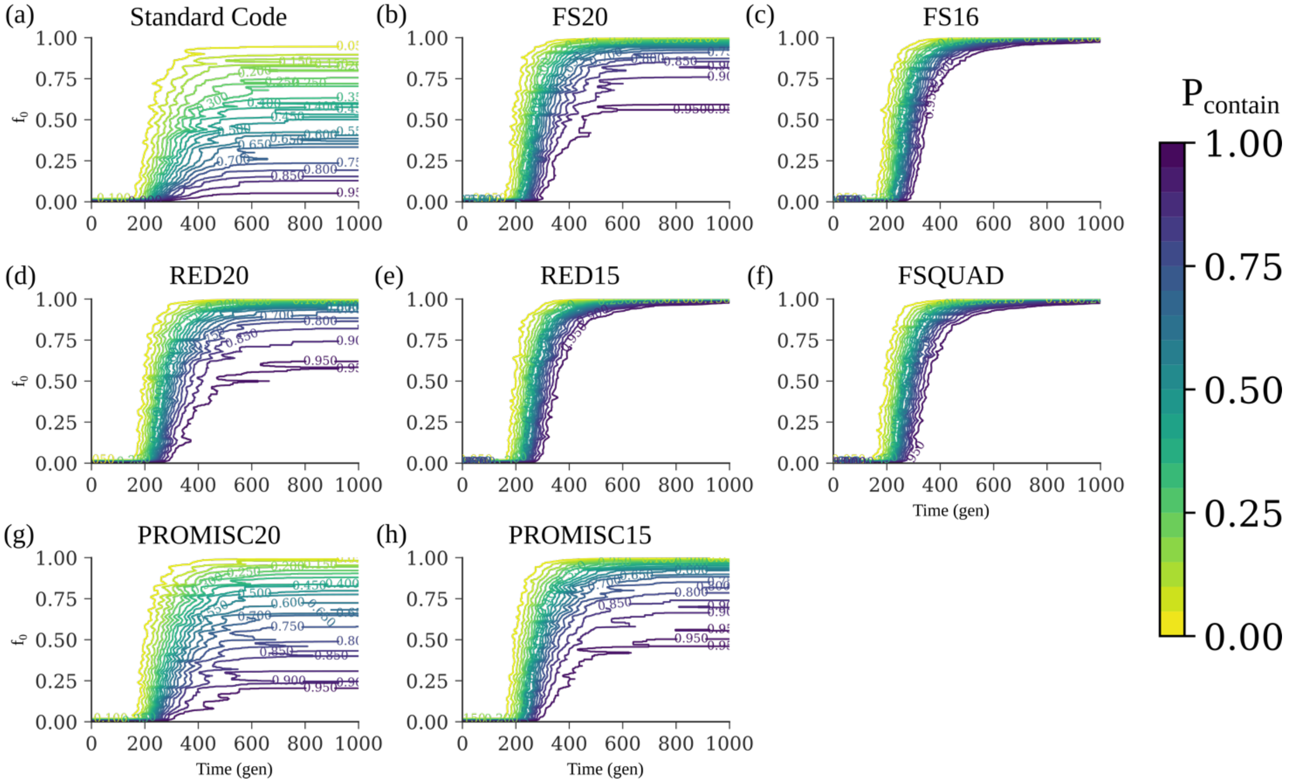

Supplementary Figure 5: Containment probability vs. initial invasive population fraction and vs. time for fail-safe genetic codes. Contour graphs of containment probability vs. time (x axis) and initial population fraction (y axis) for invasive strains using **(a)** the Standard Code, **(b)** FS20, **(c)** FS16, **(d)** RED20, **(e)** RED15, **(f)** FSQUAD, **(g)** PROMISC20, and **(h)** PROMISC15 (n = 300 replicates). Color represents $P_{contain}$ magnitude, varying from 0 (yellow) to 1 (purple). $P_{contain}$ reaches a steady state value at the limit of large *t*.

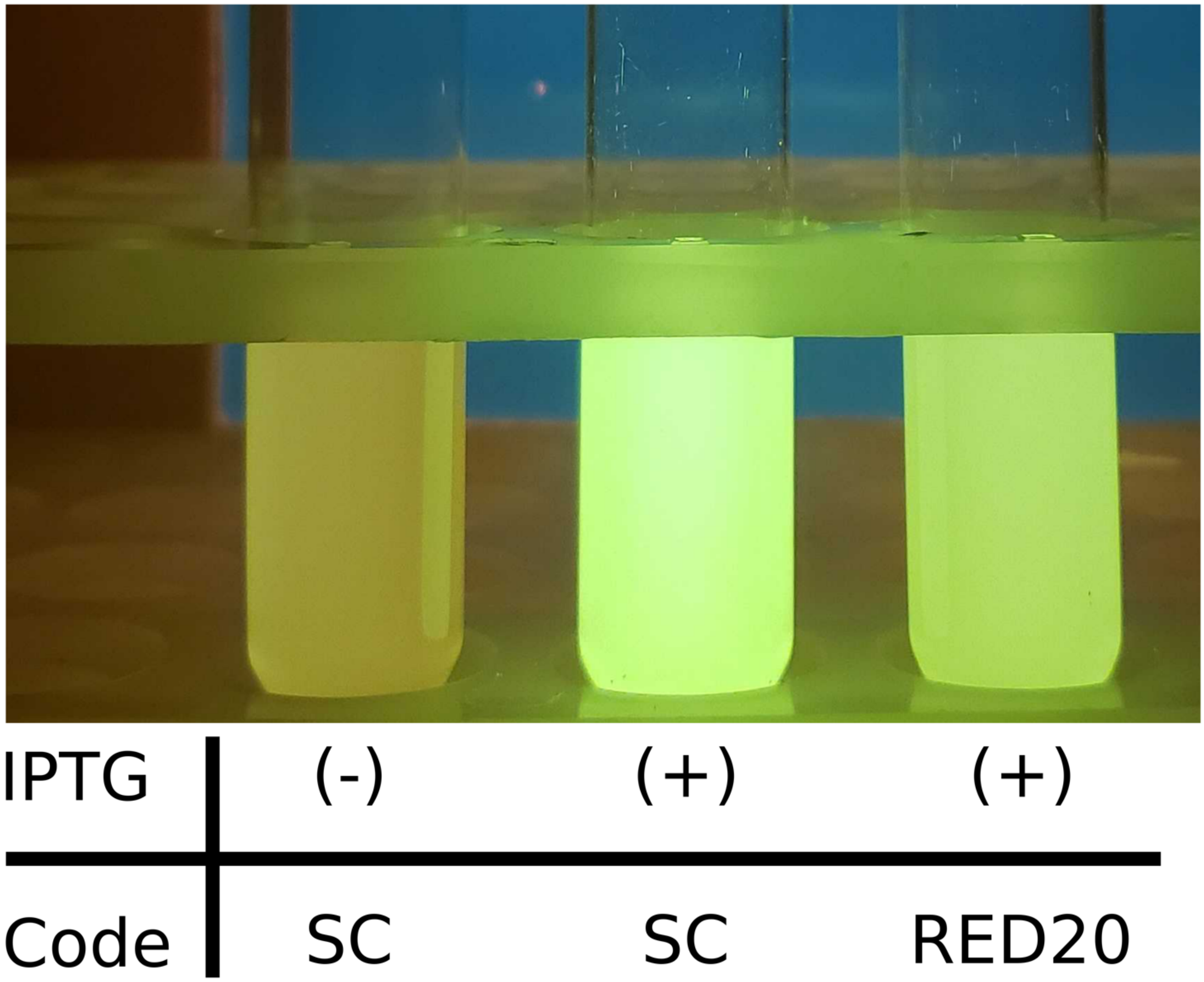

***Supplementary Figure 6: RED20-encoded fluorescent protein is expressed* in vivo:** Standard-encoded (SC) or RED20-encoded (RED20) variants of a green fluorescent reporter under the T7 promoter were transformed into the *E. coli* strain BL21 (DE3), which expresses T7 RNA polymerase under induction from Isopropyl β-D-1-thiogalactopyranoside (IPTG), and imaged under blue light three hours after induction. Uninduced cells (- IPTG) did not fluoresce, whereas induced cells carrying either reporter fluoresced (+ IPTG).

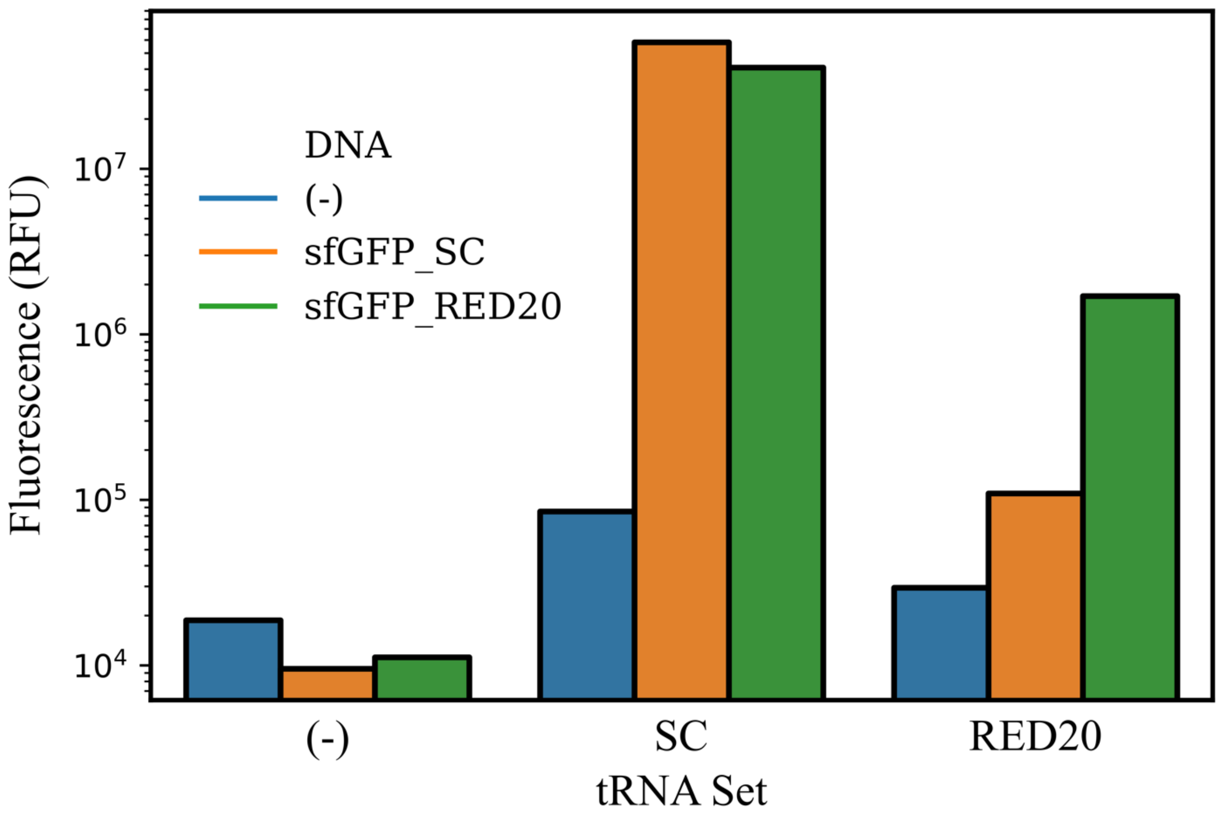

Supplementary Figure 7: PURE ΔtRNA shows fluorescence below noise floor: *In vitro* reactions were assembled using the PURE system lacking tRNAs (PURE ΔtRNA) supplemented with either control tRNAs instantiating the Standard Code (SC, middle), synthetic tRNAs instantiating RED20 (RED20, right), or no tRNA (negative, left). Reactions were run with either a Standard-encoded (sfGFP_SC, orange) or RED20-encoded (sfGFP_RED20, green) variant of a green fluorescent reporter under the T7 promoter, or with no DNA template (negative, blue), and endpoint fluorescence was measured using a plate reader after 16 hours.

Position	Amino Acid	Conservation Score	MSA	Residue Diversity
5	E	-0.145	11/46	{'P', 'A', 'E', 'R', 'K'}
6	E	-0.492	14/46	{'L', 'A', 'Q', 'E', 'K'}
14	I	0.602	23/46	{'S', 'L', 'V', 'M', 'Y', 'C', 'F', 'T', 'I'}
17	E	0.153	37/46	{'N', 'S', 'H', 'F', 'D', 'E', 'R', 'T'}
23	N	-1.222	45/46	{'N', 'G', 'H', 'D', 'E'}
25	H	-0.873	45/46	{'N', 'H', 'M', 'Q', 'K'}
32	E	1.616	45/46	{'N', 'S', 'H', 'V', 'Q', 'D', 'E', 'K', 'T'}
34	E	2.599	45/46	{'N', 'S', 'L', 'V', 'A', 'Y', 'Q', 'D', 'E', 'K', 'T', 'I', 'R'}
47	I	1.041	45/46	{'N', 'S', 'V', 'C', 'Y', 'I', 'D', 'E', 'K', 'T', 'W', 'R'}
48	C	-1.12	45/46	{'S', 'L', 'C', 'V', 'I'}
70	C	0.599	46/46	{'P', 'L', 'V', 'A', 'M', 'C', 'Q', 'F', 'I'}
77	H	0.414	46/46	{'N', 'S', 'G', 'H', 'V', 'D', 'E'}
**81**	**H**	**0.257**	2/46	**{'N', 'H'}**
90	E	-0.029	46/46	{'S', 'V', 'A', 'M', 'D', 'E', 'K', 'T'}
95	E	-0.43	46/46	{'H', 'Q', 'D', 'E', 'R', 'T'}
98	I	-0.138	46/46	{'L', 'V', 'M', 'F', 'T', 'I'}
105	N	-0.348	46/46	{'N', 'S', 'V', 'A', 'C', 'Q', 'F', 'K', 'T', 'I'}
111	E	1.798	46/46	{'N', 'H', 'V', 'A', 'M', 'Q', 'D', 'E', 'K', 'T', 'R'}
115	E	1.472	46/46	{'S', 'L', 'V', 'A', 'Q', 'D', 'E', 'K', 'T', 'I', 'R'}
121	N	-0.508	46/46	{'N', 'S', 'H', 'V', 'A', 'Y', 'Q', 'D'}
123	I	-0.936	46/46	{'S', 'V', 'F', 'T', 'I'}
124	E	0.671	46/46	{'N', 'S', 'M', 'I', 'Q', 'D', 'E', 'K', 'T', 'R'}
128	I	0.061	46/46	{'N', 'S', 'G', 'L', 'V', 'M', 'Q', 'D', 'E', 'K', 'T', 'I'}
132	E	1.541	46/46	{'S', 'P', 'H', 'L', 'V', 'A', 'I', 'D', 'E', 'K', 'T', 'R'}
135	N	-1.111	46/46	{'N', 'V', 'P', 'H'}
136	I	-1.148	46/46	{'L', 'V', 'T', 'I'}
139	H	-0.344	46/46	{'N', 'S', 'H', 'M', 'Q', 'D', 'R', 'K'}
142	E	2.252	46/46	{'H', 'L', 'V', 'A', 'Y', 'M', 'D', 'E', 'T', 'I'}
144	N	0.379	46/46	{'N', 'S', 'H', 'G', 'M', 'Q', 'D', 'E', 'K', 'R'}
146	N	-0.378	46/46	{'N', 'P', 'H', 'L', 'Q', 'D', 'E', 'R', 'T'}
148	H	0.349	16/46	{'N', 'S', 'H', 'G', 'V', 'Q', 'D'}
149	N	0.611	46/46	{'N', 'S', 'L', 'V', 'A', 'Y', 'M', 'Q', 'F', 'C', 'E', 'T', 'I'}
152	I	-0.405	46/46	{'L', 'V', 'A', 'M', 'T', 'I'}

159	N	0.157	46/46	{'N', 'S', 'H', 'G', 'D', 'E', 'K', 'T'}
161	I	-0.707	46/46	{'L', 'V', 'M', 'I'}
164	N	0.72	46/46	{'N', 'S', 'H', 'L', 'V', 'A', 'Y', 'F', 'D', 'E', 'I'}
167	I	-0.289	46/46	{'N', 'V', 'A', 'Y', 'M', 'Q', 'F', 'I', 'K', 'R', 'W'}
169	H	-0.241	46/46	{'H', 'Y', 'L', 'F'}
170	N	0.677	46/46	{'N', 'S', 'P', 'L', 'V', 'M', 'Q', 'D', 'E', 'K', 'T', 'R'}
171	I	-0.591	46/46	{'L', 'V', 'K', 'T', 'I'}
172	E	0.613	46/46	{'N', 'V', 'A', 'M', 'Q', 'E', 'K', 'T'}
181	H	-0.432	46/46	{'N', 'S', 'H', 'C', 'V', 'Q', 'F', 'T', 'I'}
185	N	0.026	46/46	{'N', 'H', 'G', 'L', 'V', 'A', 'M', 'E', 'K', 'R'}
188	I	0.947	23/46	{'N', 'G', 'L', 'V', 'D', 'I'}
198	N	1.292	45/46	{'N', 'S', 'P', 'V', 'A', 'Y', 'F', 'T', 'W'}
199	H	-1.559	45/46	{'V', 'Y', 'H'}
212	N	1.277	39/46	{'N', 'S', 'L', 'Q', 'D', 'E', 'K', 'T', 'R'}
213	E	-0.907	37/46	{'D', 'E', 'A', 'S'}
217	H	-0.337	27/46	{'N', 'H', 'L', 'V', 'F', 'D', 'K'}
222	E	-0.597	25/46	{'H', 'D', 'E', 'R', 'K'}
229	I	0.228	7/46	{'H', 'V', 'A', 'T', 'I'}
231	H	-0.519	4/46	{'G', 'H'}
235	E	0.318	3/46	{'L', 'E'}

Supplementary Table 2: ConSurf analysis of residue conservation in fluorescent variants of GFP. Positions in GFP encoding amino acids omitted from RED15 including position number (Position), consensus amino acid (Amino Acid), ConSurf conservation score (Conservation Score), number of variants in the multiple sequence alignment with an amino acid at the listed position (MSA), and all amino acids found at that position across fluorescent GFP variants (Residue Diversity) [Ashkenazy *et al.* 2016]. All positions show a diversity of encoded amino acids across fluorescent variants which include at least one amino acid in the RED20 supported amino acid set, except for except 81H (bolded). 81H however is only found in two of 46 variants in the multiple sequence alignment.

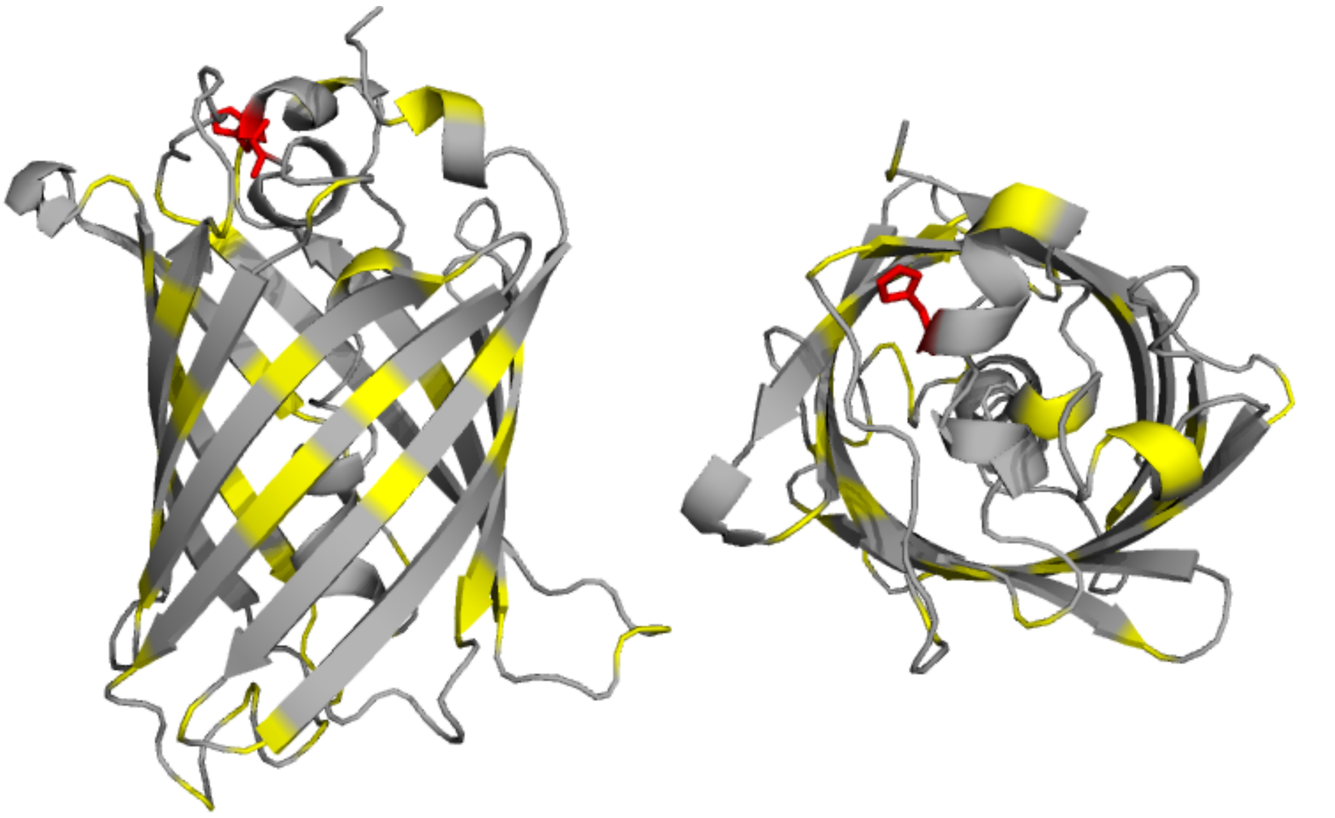

Supplementary Figure 8: GFP structure with positions using non-RED20 amino acids highlighted.

Side (left) and top-down (right) views of GFP crystal structure (PDB: 1gfl) [Yang, Moss, and Phillips Jr. 1996]. Positions in which the canonically encoded amino acid is omitted from RED15 are marked in yellow. H81, the only position for which the residue diversity at the position lacks any amino acid in RED15, is marked in red.
